# Primus Inter PARES: First among equals—practical strategies for young adult PAtient RESearch partners (PARES) by young adult PARES

**DOI:** 10.1186/s40900-024-00576-0

**Published:** 2024-05-08

**Authors:** Sandy Rao, Gina Dimitropoulos, Rae Jardine, Julien Quickstad, Laetitia Satam, Mohammad Qureshi, Thyra Bui, Antoaneta Alexandrova Todorova, Ysabelle Tumaneng, Abitha Suthakaran, Kaiden Dalley, Stacie Smith, Scott B. Patten, the entire HEARTS Study Team

**Affiliations:** 1 Faculty of Social Work, University of Calgary, Calgary, Canada; 2 Mental Health Accessibility and Policy Solutions Lab, Mississauga, ON Canada; 3 Department of Psychiatry, University of Calgary, Calgary, Canada; 4 Mathison Centre for Mental Health Research & Education, University of Calgary, Calgary, Canada; 5 University of Alberta, Edmonton, AB Canada; 6 Dalla Lana School of Public Health, University of Toronto, Toronto, Canada; 7 Faculty of Education, Mount Saint Vincent University, Halifax, NS Canada; 8 Department of Community Health Sciences, University of Calgary, Calgary, Canada

**Keywords:** Patient research partners, Patient-oriented research, Canada, Young adults, Mental health, Equality, Trauma-informed, Reciprocity, Meaningful engagement, Compensation

## Abstract

**Background:**

This manuscript is coauthored by 15 young adult Patient RESearch partners (PARES) with lived and living mental health experiences and three institutional researchers across Canada involved in a patient-oriented research (POR) study called the HEARTS Study: Helping Enable Access and Remove Barriers To Support for Young Adults with Mental Health-Related Disabilities. We share our reflections, experiences and lessons learned as we grapple with the field of POR for its lack of clarity, hierarchical structures, internalized ableism, and accessibility challenges, among others. To mitigate the difficulties of POR, we started by laying the groundwork for equality by embracing the principle of *Primus Inter Pares: First Among Equals* as the foundation of our approach. In this way, we began with what we know for certain: the inherent worth and dignity of young adults as equal partners, recognizing their expertise, worldviews, creativity, and capacity to contribute meaningfully and intentionally to the research that affects their lives and futures.

**Main Body:**

The manuscript underscores the need to reconceptualize meaningful engagement in POR, advocating a shift from traditional, biased paradigms that fail to address the complexities faced by young adults with mental illness. It introduces what we have termed *Adaptive and Differential Engagement*, underscoring the necessity of tailoring participation to individual preferences and circumstances alongside a *Tripartite Compensation model* that promotes fair and holistic remuneration in research collaborations. Then we discuss the approaches we have conceptualized, such as *Equitable Dialogue, Trust Architecture, Community Continuum, Unity in Diversity, Shared Stewardship, and Agile Frameworks* that collectively aim to overcome barriers like language intimidation, power imbalances, framework fatigue, consultation burnout, trust deficits, and systemic discrimination and exclusion. The manuscript does not seek to prescribe any universal or standardized solutions; in fact, it seeks the opposite. Instead, it offers a thoughtful and transparent contribution to the POR canon, providing resources for young adults eager to engage in research and institutional researchers aspiring to collaborate with them.

**Conclusion:**

This manuscript is a product of our collective learning and critical self-evaluation. By integrating theoretical insights with practical strategies, we present a justice-oriented blueprint for an inclusive and egalitarian approach to POR. We advocate for applications of POR that are responsive to the individualized contexts of young adult PARES, ensuring their perspectives are central to the research with the resources to take the lead should they choose.

**Supplementary Information:**

The online version contains supplementary material available at 10.1186/s40900-024-00576-0.

## Plain Language Summary

Together with a graduate student, co-supervisors, and 15 young people from across Canada who have experience with mental health challenges, this paper looks at how research involving young adults as patient research partners can be better. We feel that the types of research that are supposed to include us are often not clear enough and make it hard to join, especially those with unique life situations and health issues. So, in this paper, we suggest a few new ways of doing things where everyone is treated equally. We call it *Primus Inter PARES: First Among Equals*. This means young people are just as important as anyone else in research.

We want to change the old ways that do not consider our unique experiences as young people with mental health issues. We came up with new ideas like 'Equitable Dialogue' and 'Trust Architecture' to ensure everyone understands the research and feels that they can trust the process. We also suggest ways to make sure different voices are heard and that everyone has a fair chance to contribute.

We do not just offer a one-size-fits-all solution; instead, we share many ways to improve research to help young adults who want to be part of research and for the researchers who want to work with us. Our paper is about making research fair and including everyone's point of view. We hope this will make the research better for everyone, especially for young adults.

**Supplementary Information:**

The online version contains supplementary material available at 10.1186/s40900-024-00576-0.

## Background

The necessity for young adults' engagement in mental health research is well-established [31, 41]. However, there is a notable lack of published literature examining the impact of personal and environmental factors on the participation and quality of life of young adults with mental health conditions in patient-oriented research (POR) [67]. In the following paragraphs, the use of "we" specifically refers to the young adult patient research partners (PARES), separate from the institutional researchers. As young adults facing our own mental health challenges, there is an irony in stepping into the roles of PARES. We are navigating intense pressures and uncertainties at this time of life [3, 45], *and* we are asked to lend our voices and experiences to the very studies aimed at solving these issues.

Reflecting critically, it is clear our involvement is crucial, but it also requires careful consideration of the increased temporal, emotional and cognitive demands. We must balance our well-being with the desire to contribute, ensuring we are not overburdened in the pursuit of advancing mental health research. Young people are at a unique and complex juncture in their lives: navigating identity formation, educational advancement, relationship development, and family and community responsibilities. This complex reality requires a thoughtful approach to create opportunities for meaningful participation [37] of young adults in mental health research [23, 40].

We became PARES because we recognize our involvement can be enriching; however, we believe it is essential to acknowledge and consider theadded pressures in research planning. The barriers to participation are manifold, including intimidation by the professional echelon, lack of confidence, insufficient support, and previous experiences of unresponsive engagements [58]. Moreover, POR, as highlighted in a recent scoping review requires careful execution since it carries real and documented risk of harm if not conducted thoughtfully [39]. For instance, psychological distress may arise if PARES recount traumatic events without adequate support or if their input is minimized or disregarded. Furthermore, there is a risk of exploitation when PARES are inadequately compensated for their expertise, or their efforts are leveraged solely to advance researchers' careers without due recognition or benefit to the PARES community [54].

### Turning theories, models, and frameworks into action: a practical driven approach for engaging in participatory research

Amidst a deluge of participatory research frameworks, models, and theories—of which 202 were identified [56], participatory research is mired in a paradoxical state where increased information has led, in the team’s opinion, to oversaturation and diminished clarity, plunging researchers into a quagmire of cognitive overload and literature review fatigue. Central to the discussion of the myriad of frameworks is the concept of *meaningful* patient engagement, a term frequently mentioned in literature yet lacking any precise or agreed-upon definition.

Meaningful patient engagement is typically associated with the more interactive levels of engagement, for example, involve, collaborate, and partner; conversely, the less interactive levels of inform and consult are often considered to represent less meaningful forms of patient engagement [37]. Within the sphere of patient engagement, POR has emerged as a distinct strategy in Canada. POR is characterized as a continuum of research that not only involves patients as research partners but also concentrates on priorities identified by them to enhance patient outcomes [10, 30].

As a team, our primary concern with the rapidly emerging scholarly discourse in POR; it threatens to diverge from its community-centric origins aimed at social improvement. We champion the "Southern tradition" of participatory research born from the struggles of equity-deserving communities fighting oppression and shaped by global scholars such as Paulo Freire. The essence of this participatory approach is diminished when academic institutions appropriate its methods without preserving the original liberatory intent. This misalignment risks turning a once-radical tool for societal transformation into a subtle form of control, thereby perpetuating the very dynamics it was created to dismantle. If the academic community does not actively uphold the emancipatory principles of participatory research, we risk diluting its potential as an agent of genuine change and reducing it to a tokenistic exercise [5, 16, 46, 49, 63].

What once was a collective endeavour is now at risk of devolving into an exclusionary, elitist academic pursuit. This shift has erected daunting barriers, rendering participatory research a privilege of the few rather than a right of us all. If this trend continues unchecked and unaddressed, we risk witnessing the erosion of authentic participatory research, leaving it as a relic within academic echo chambers, disconnected from the communities it was meant to serve. Hillier et al. [24] confirm as POR becomes more mainstream, there is a necessity for clear mechanisms to support the inclusion of PARES in health research.

POR, while valuable, has been observed to be complex, time and resource-intensive and often theoretically-laden [6, 48, 64]. The complexity becomes even more pronounced for young adults with mental illness interested in participating as PARES who are also at a critical juncture in life, grappling with a multitude of pressures and responsibilities [21, 27, 38]. Without thoughtful consideration, engaging young adults with mental illness as partners in research risks exacerbating normative challenges of young adulthood, potentially compromising the young adults and the research [52].

Given these concerns, our team is applying approaches to POR that transcend traditional paradigms, aiming instead to address previously documented challenges of POR through the lens of social justice and equity. This paradigm shift calls for a committed and empathetic exploration into the lives of young people—*by young people*, grounded in the principles of justice, democracy, and human rights. In doing so, we collectively affirm our commitment to building a society that values and supports its young, recognizing them as equal partners in the journey toward a more just future [23].

As such, the goals of the manuscript are:

1. Advance an Authentic and Transparent POR Narrative: We strive to share our real-time experiences navigating POR with humility and openness. Our goal is to contribute to a more transparent and authentic discourse in POR, reflecting our journey with honesty.
2. Defend the Democratic Roots of POR Against Academization: A critical objective of our work is to preserve the democratic and egalitarian principles that form the bedrock of participatory research. As POR increasingly integrates into the academic domain, we aim to maintain its foundational community-centric values and resist the shift toward academization [25].
3. Transform Barriers into Breakthroughs: Acknowledging the imperfections in the current state of POR, our manuscript seeks to demonstrate how we utilize available resources and insights to overcome challenges and progress within the field.
4. Implement Tailored Approaches for Young Adults with Mental Illness: Recognizing the specific needs of young adults with mental health conditions, we aim to highlight the importance of a nuanced and empathetic approach in conducting POR with this demographic.

Our overarching objective in disseminating this manuscript is to demystify POR, making it more accessible and less daunting for both institutional researchers and PARES. Through this manuscript, we aim to showcase POR as a practice that is not only achievable but is also enriched by the diverse contributions and perspectives of all involved, in line with the ethos of Primus Inter PARES.

### Discourses, language, positionality and representation of voice

#### *Mental health, young adults and discourses *of *vulnerability*

We acknowledge that terms such as “mental illness," “mental disorder," and “patient” are fraught with challenges [2, 9]. However, we use this language for practicality’s sake throughout the manuscript. Moreover, we avoid the term 'vulnerability' due to its ambiguous and potentially problematic nature. This decision stems from a recognition that terms such as 'vulnerable,' 'marginalized,' 'disadvantaged,' 'at risk,' 'underserved,' and 'disenfranchised' often lack clear definitions, leading to potential misunderstandings and implications of intrinsic deficits within certain populations [29, 44, 53].

Additionally, the use of 'vulnerable' can obscure the broader structural and societal factors contributing to these challenges, concealing the structural causes of health inequities and reducing accountability for these disparities. Instead, where required, we attempt a more inclusive approach, identifying specific health-related circumstances that increase the risk for vulnerability rather than broadly labelling young adults with mental illness and related impairments and disabilities as ‘vulnerable’ [60].

#### Context and locatedness: group positionality statement and representation of voice

This manuscript is a collaborative effort involving 15 young adult PARES and three institutional researchers across Canada, representing the ten Canadian provinces. The PARES have personal experiences with mental illness, and the institutional researchers—a graduate student and co-supervisors—bring expertise in mental health, youth research, and participatory methods. Together, we are a co-researcher team on a POR study called the HEARTS Study (Helping Enable Access and Remove Barriers To Support for Young Adults with Mental Health-Related Disabilities) focused on young adult mental health impairments and disabilities and access to mental healthcare. Our study defines young adults as aged 18–30. The World Health Organization [65] notes that while age provides a useful framework for delineating periods of development, particularly for marking biological events, it is less indicative of social transitions, which are heavily influenced by sociocultural factors. The 18–30 age range reflects the increasingly varied and extended transitions between adolescence and full independence, which is increasingly defined as ending later [48].

In the HEARTS Study, the PARES are engaged as coresearchers, directly involved in every stage of the research, including study design, recruitment of study participants, collecting and analyzing data, writing academic publications and contributing to the dissemination of findings through knowledge mobilization activities. The institutional researchers, alongside the research activities named above, provide guidance, supervision, funding and resources support, mentorship, and administrative support and ensure the research maintains academic and methodological integrity. This integrative approach ensures that the lived and living experiences of PARES inform all aspects of the study, from conception to completion.

Our research team includes members ranging from novices to seasoned researchers. Moreover, our range of mental health experiences, both personal and familial, offers a spectrum of experiential knowledge that directly informs our research perspective and approach. The diversity of our research team is multifaceted, encompassing a wide array of cultural, 2SLGBTQI + and socioeconomic backgrounds, as well as lived experiences with physical disability and chronic health conditions. Our cultural diversity includes South Asian (Sri-Lankan, Punjabi), Vietnamese, Ukrainian/French Canadian, Filipino/ Filipinx, Balkan, Indigenous, Acadian, and individuals from the Black diaspora. Our team’s gender and sexual identity diversity includes cisgender, Transgender, Two-Spirit, Genderqueer, Gay, Bisexual, and Queer identities. Our lived experiences encompass those of physical disabilities, neurodivergence, addictions, and chronic health conditions.

Our team members' experiences range from economically advantaged to navigating precarious housing situations with limited communication infrastructure. Team members are spread between urban centres and rural locals with diverse living arrangements and family compositions. By utilizing our collective plurality, we challenge the conventional norms of research and advocate for a more inclusive and responsive healthcare research landscape with the ultimate goal of improving how healthcare systems serve individuals and communities. The manuscript most often reflects the collective voice of the entire HEARTS Study team; however, where there are sections authored solely by PARES and reflect an unfiltered narrative of their collective experiences and insights, distinct from those of the institutional researchers, that will be indicated in the text.

## Our process of non-traditional manuscript-making

In crafting this manuscript, we pursued a unique approach emphasizing the active participation of all PARES, underscoring our commitment to transparency and collaborative ethics. This process, developed in collaboration with the HEARTS study ethics officer, graduate student (GS), and co-supervisors, was guided by an ethical framework addressing the complexities of shared authorship and equitable distribution of credit. Importantly, our methodology was akin to reverse engineering; instead of converting academic manuscripts into plain language summaries, we distilled our collective experiences, discussions, and deep engagement with the literature into an accessible format before translating it into academic language.

**Fig. 1:**
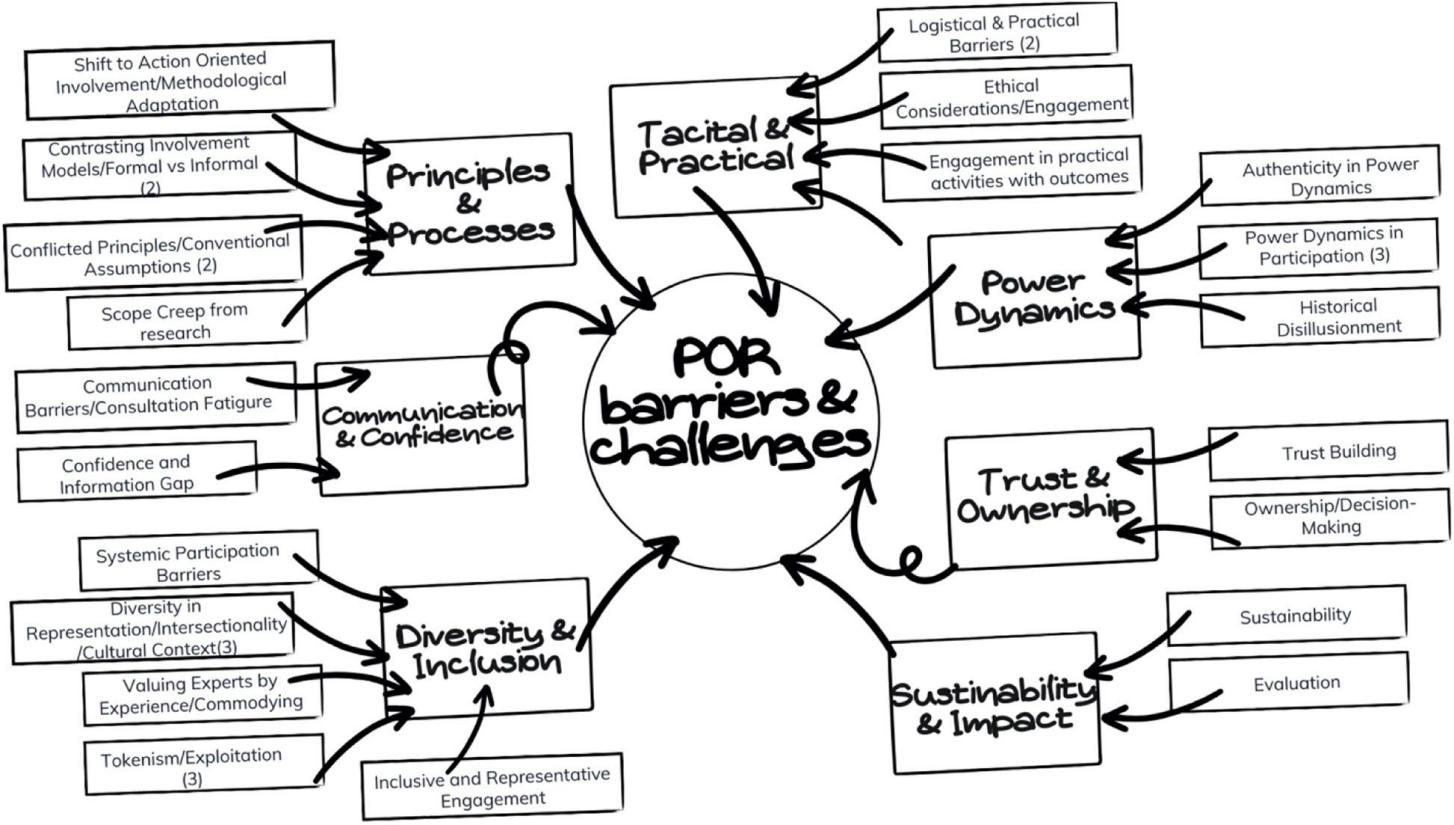
Patient-oriented research barriers and thematic synthesis

Over 180 h of individual consultations via digital platforms allowed us to accommodate the varied experiences of PARES, some engaging in research for the first time. The GS synthesized an extensive literature review and annotated bibliography into simplified, theme-based summaries, facilitating a shared foundational understanding of prior research on POR that was necessary for collaboration (see Fig. 1). Through weekly Writers' Circles—an hour-long meeting where we shared and refined our manuscript drafts through peer-to-peer feedback and mentorship, PARES contributed their unique insights and deconstructed the literature through reflection on their lived experiences.

**Fig. 2:**
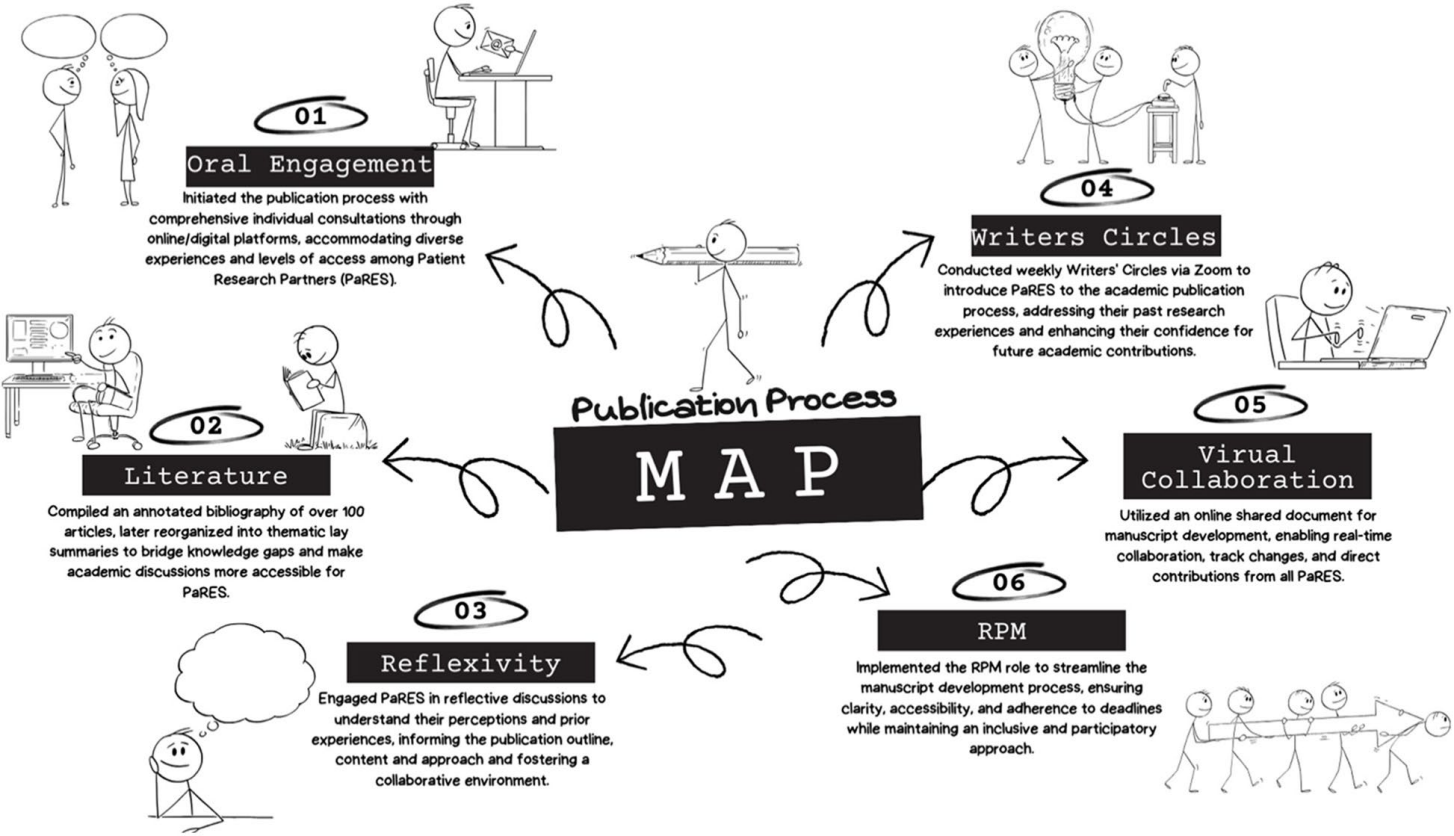
Our publication process map

The collaborative manuscript writing process highlighted the need for structured management to handle the volume of collaborative input. Consequently, we introduced the Rotational Publication Project Manager (RPM) role, initially undertaken by the GS, to oversee administrative aspects and maintain an organized framework for effective participation. This role was crucial in ensuring that the diverse contributions of all team members were coherently integrated into the manuscript. For an in-depth explanation of our non-traditional manuscript-making process (Fig. 2), including the specifics of our reverse engineering approach and the contributions of the PARES, please refer to the Supplementary material.

## Main text

### Primus Inter PARES

In our exploration of the literature, we discovered a pivotal truth: the culture and values that underpin a POR study are significant determinants of its success, often having a greater impact than the intricacies of methodologies or frameworks [6, 14, 15, 22, 61]. This insight deeply resonated with us in our prior experiences, revealing that the most substantial issues in research often stem not from explicit procedural faults but from subtler aspects embedded within the research environment. These cultural elements, although intangible, profoundly shaped our engagement and perceptions, highlighting the critical role of the values and ethos upheld by institutional researchers and the structures and research traditions they subscribe to.

Guided by these findings, we have embraced the principle *Primus inter PARES*, or *First among Equals*, [8, 19] to set the tone and culture of our research. This Latin axiom is foundational to the HEARTS Study, ensuring that all individuals are treated as equal partners, irrespective of their prior formalized *research* expertise and practice-based experience. We must clarify that ‘equal’ does not mean ‘same;’ rather, it acknowledges that while different individuals bring varied subject matter expertise, each contribution is equally vital to our *collective pursuit of knowledge*. This approach reflects Kenneth Blanchard's doctrine that "None of us is as smart as all of us," [8] emphasizing the importance of harnessing and cultivating collective wisdom.

Further aligning with this ethos, we intentionally chose the term 'PARES.' While serving as an acronym for PAtient RESearch Partners, it also parallels the Latin 'PARES,' meaning 'equals.' This nomenclature symbolizes our dedication to fostering an environment where mutual respect and reciprocity are paramount. Such an egalitarian stance in mental health research is essential, ensuring that our research processes are inclusive, respectful, and fundamentally rooted in equality. This approach affirms our commitment to creating a research space where every individual’s voice is not only heard but is integral to the progression and success of our research endeavours.

### Adaptive and differential engagement: redefining ‘Meaningful’

As a cohesive research team, our journey in conducting mental health research is an exercise in capacity sharing and harnessing the multifaceted strengths inherent in our group. We feel that the conventional notion of 'meaningful engagement' in POR, as mentioned above, harbours unspoken biases and privileges influenced by a blend of ableism (discrimination against people with disabilities), the dynamics of capitalism (how the economic system of private business ownership operates and evolves), and the Protestant work ethic (the belief that hard work and discipline are moral virtues) [1, 7, 13, 35].

**Fig. 3:**
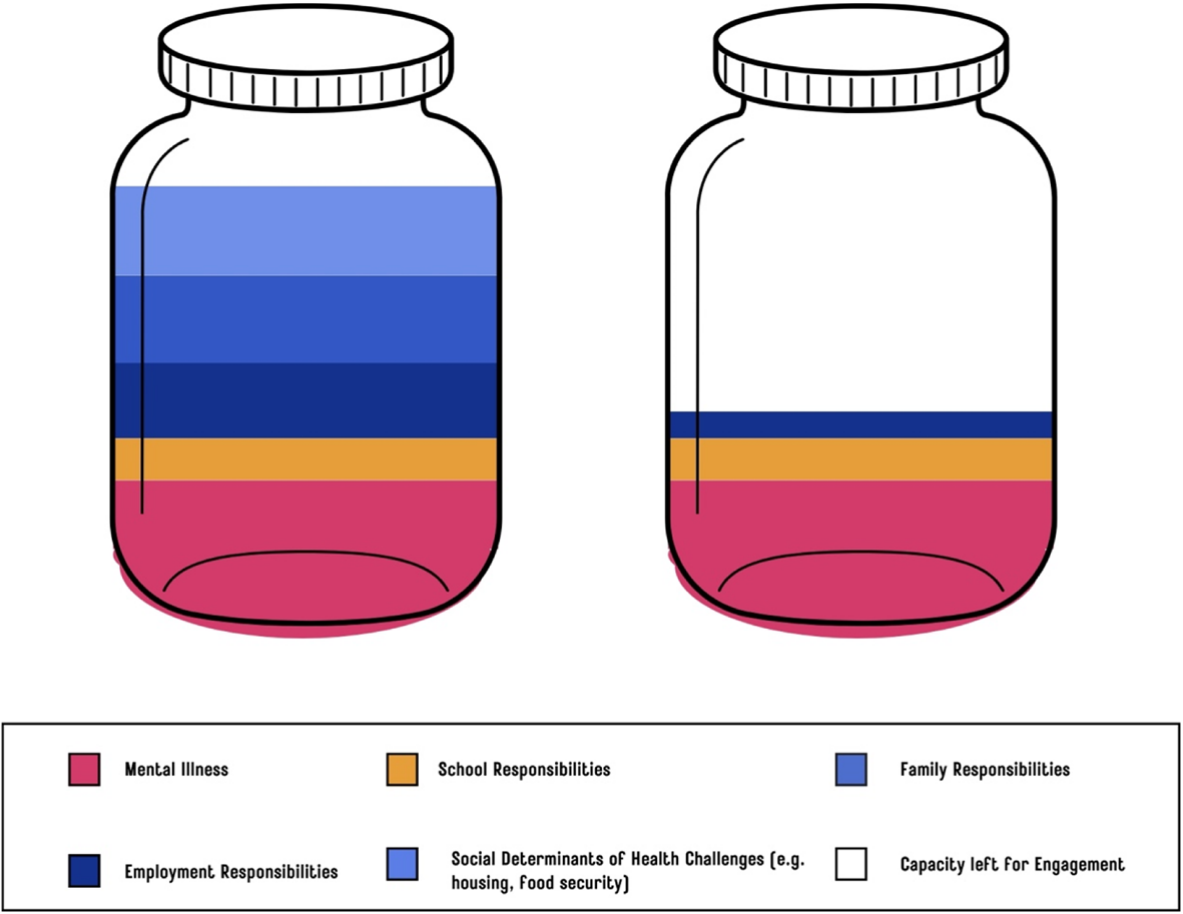
Variability in young adults' capacity for engagement due to life and health circumstances, systemic and structural barriers

Such a view may inadvertently overlook the realities of PARES juggling demanding jobs, academic deadlines, family responsibilities, or personal health issues and thus their resulting capacity to engage (Fig. 3). We challenge traditional perceptions of engagement, recognizing that involvement intersects with structural and systemic barriers [43]. Instead, we understand meaningful engagement as a spectrum of contributions where less visible but significant efforts are equally valued [57].

We propose an alternative approach, *adaptive and differential engagement*. 'Adaptive Engagement' is characterized by its flexible and responsive nature. This aspect encourages bespoke strategies tailored to the individualized and evolving contexts of PARES and institutional researchers, facilitating their participation in a manner that respects and adapts to their specific needs and capabilities. 'Differential Engagement' acknowledges the diverse forms and levels of engagement that emerge due to personal, social, and health-related factors. This principle underscores our understanding that engagement and participation will naturally differ among individuals *and* along different time points of the project, and these differences are not simply accommodated but are deeply valued as integral to the richness and inclusivity of our research. By adopting these considerations, we aim to create a research environment that is equitable, empathetic and open to continuous change, ensuring that our practices are deeply rooted in the real-world experiences and contributions of all team members, especially those navigating the complexities of mental health challenges.

### Tripartite compensation: advocating for equitable remuneration in research

Speaking as the PARES, we are keenly aware of the issues surrounding compensation in POR [34, 50, 51]. In many of our experiences, discussions around financial remuneration in research settings appear shrouded in a cultural veil of taboo. We feel addressing compensation openly is vital for challenging entrenched power dynamics and advancing the cause of economic justice in research collaborations. Embracing the principle of Primus Inter PARES, we stand firm in our belief that involvement in research should not lead to financial detriment for any PARES.

**Fig. 4:**
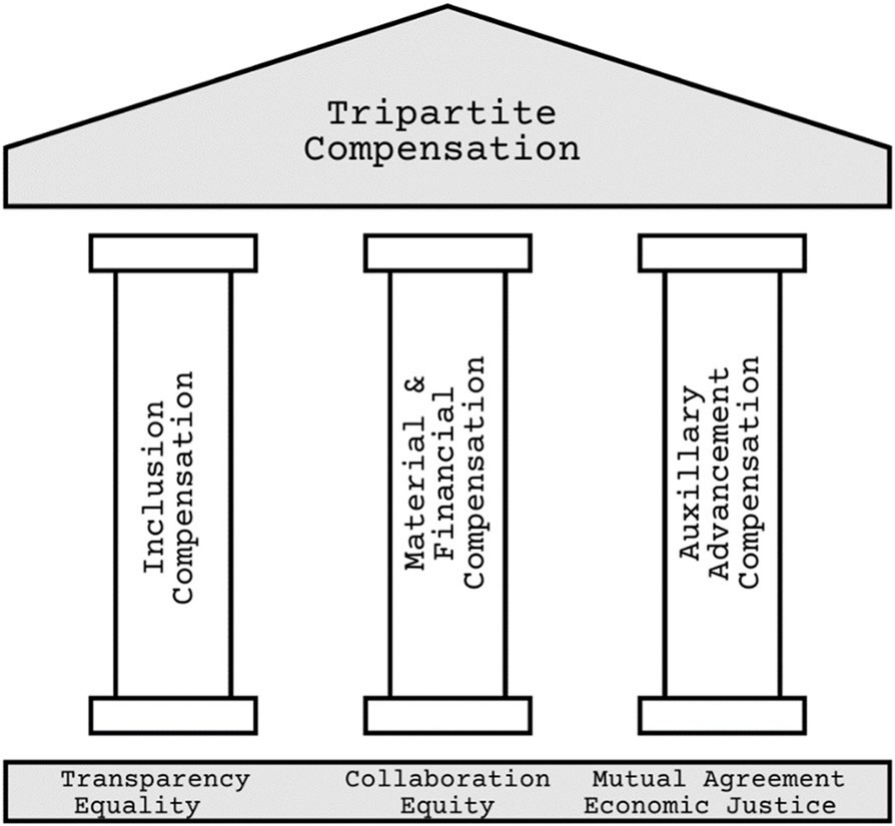
Tripartite Model of Patient-Oriented Research Compensation

As a team, through our experiences and dialogues, we have identified three distinct yet interconnected forms of compensation, often overlooked or insufficiently differentiated in academic discourse. Recognizing these forms is key to developing practical, nuanced recommendations for PARES remuneration. We also recognize that while some may *choose* minimal material compensation, perhaps driven by altruistic motives, this is not a feasible *or fair* option for all. Thus, we challenge the reductionistic view of research participation as purely altruistic or financially motivated, intrinsic or extrinsic, and suggest a position that subsumes all. Below we define the pillars of our tripartite compensation model (Fig. 4).

#### Inclusion Compensation

In our first pillar of compensation, 'inclusion compensation,' we acknowledge the different starting points of PARES. This compensation is a step towards levelling the playing field, particularly for those lacking the privileges often associated with wellness and socio-economic stability [33, 62, 66]. It includes essential supports like reliable internet, computing devices, financial aid for easing life stressors, childcare services, educational credits, and transportation solutions. Our aim here is to enable wider participation in research, moving beyond a model favouring only those who can afford to engage without additional support.

#### Material or financial compensation

Our second pillar, 'material or financial compensation,' goes beyond the traditional focus on compensating for the lived experience of health conditions. We believe in recognizing the full array of contributions we bring to research beyond *just* our health experiences. For example, in today's digitally driven world, many of us bring invaluable expertise in areas like social media and digital content creation, which may be essential for many aspects of the research process. Moreover, we feel it is crucial to acknowledge that as the demand for POR intensifies [63], institutional researchers are becoming more dependent on the involvement of PARES to facilitate the possibility of POR. This transition underscores a power shift, recognizing PARES not merely as props but as essential collaborators whose engagement is essential.

As PARES, our commitment to POR signifies a considerable dedication of time and effort that often precludes other personal and professional pursuits. The reality of this engagement means that we might forego additional employment, limit interactions with friends and family, or reduce the time allocated to school. Therefore, compensation should fairly reflect our diverse expertise and the personal sacrifices involved in our participation [34].

#### Auxiliary advancement compensation

The third pillar, ‘auxiliary advancement compensation,' involves the options often provided for academic and professional development, such as contributing to publications and participating in conferences. Honest provision of these opportunities requires careful resource allocation, covering costs like publication fees, conference expenses and support mechanisms such as compensatory time off work or childcare services. This ensures that these opportunities are genuinely accessible and beneficial to all of us and not just PARES with expendable income or institutional researchers with hefty research grants.

Implementing this tripartite compensation model is guided by transparency, collaboration, and mutual agreement. It necessitates an understanding of PARES unique needs and preferences with institutional researchers, alongside systems and structures, adapting compensation strategies accordingly. This includes considering the impact of payments on educational or social assistance benefits and ensuring that participation does not worsen PARES’ financial situation. We advocate for various payment methods, including e-transfers, gift cards, lump sums, intermittent payments, bill payments, or formal employment with pre-deducted taxes. Continuous review and adaptation of these strategies are paramount to maintaining ethical integrity and economic justice and effectively responding to evolving needs and the changing research landscape.

**Fig. 5:**
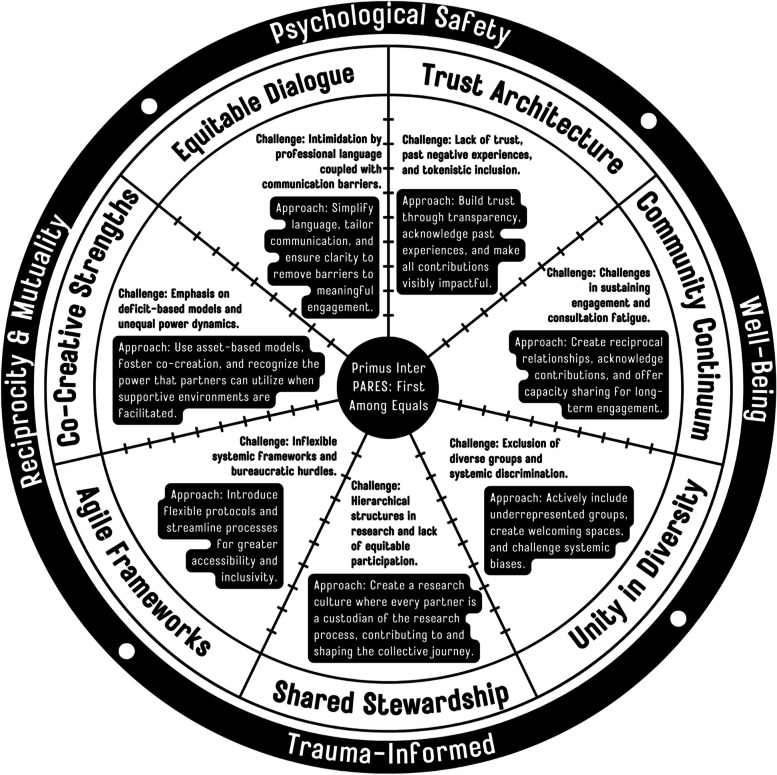
Navigating Challenges in Patient-Oriented Research: A Strengths-Based Approach for Equality, Inclusivity and Justice-Based Engagement

In the following section, we move from issues to solutions, detailing our strengths-based co-creative approaches to move from theory to practice; we discuss first our approaches to dealing with the major challenges and barriers to POR, and then we provide the application and implementation of those approaches along with materials and methods that may be required and considerations for implementation. Figure 5 provides a visual overview.

### From barriers to breakthroughs: young adult PARES’ guide to POR

The intimidation caused by professional language in POR often acts as a barrier to engagement [40, 52, 58]. To counter this, our *Equitable Dialogue* strategy advocates for the simplification of language to facilitate clear and inclusive communication. This approach is designed to ensure that each PARES, irrespective of academic expertise, is able to contribute.Trust within the research context is frequently undermined by previous negative experiences [40, 48, 55, 58]. Our *Trust Architecture* approach aims to rebuild this trust by fostering a transparent and consistent environment. This method underscores the importance of making each PARES contribution visibly impactful, reinforcing their significance to the project’s success.

A common issue in POR is the difficulty of sustaining long-term engagement [4, 48]. Our *Community Continuum* strategy addresses this by creating a culture of mutual support and continuous collaboration through mentorship, capacity-sharing and community and relationship building. This ensures that the collaborative spirit endures beyond the project’s lifespan. The exclusion of diverse groups and the presence of systemic discrimination [4, 15, 35, 42] are challenges we tackle with our *Unity in Diversity* approach. This strategy ensures the intentional inclusion of underrepresented groups in our research team, study, process and narrative, fostering a setting that celebrates diverse strengths and contributions while actively working against discrimination and stigma.

Hierarchical structures in research can create power imbalances that hinder equitable participation [11, 17, 18, 26, 32]. To address this, we introduce *Shared Stewardship*, a model that promotes a non-hierarchical, collaborative research environment. This framework emphasizes shared decision-making and values the contributions of each individual equally.

The proliferation of complex frameworks within the academic domain frequently manifests as a formidable barrier rather than an inviting landscape for prospective researchers, partners and participants [4, 37, 48, 56, 63, 64]. This metaphorical “academic fortress,” as some of us prefer to call it, hinders the openness and approachability necessary to engage with POR. Our *Agile Framework* approach helps us to dismantle these metaphorical walls and distill and synthesize the essence of these multiple frameworks into a more digestible and PARES-friendly format. This consolidation is intended not only to facilitate entry into POR but also to make the process of engagement more intuitive and less daunting.

By refining procedures and enhancing technological interfaces, we aspire to create a more navigable and inclusive environment that welcomes and supports all PARES. Moreover, the subtext of deficit-based models in research can perpetuate unequal power dynamics for PARES [15, 28, 29, 35, 44, 48, 64]. Our *Co-Creative Strengths* approach counters this tendency by focusing on the inherent strengths of all PARES, fostering an empowering and balanced research environment.

### Well-Being, reciprocity and mutuality, trauma-informed and psychological safety

The concept of well-being is pivotal to our approach, particularly in the context of young adults with mental health experiences engaged in participatory research. Aligned with the World Health Organization’s definition, well-being is acknowledged as a fundamental component of health, encompassing the ability to realize one's potential, cope with everyday stresses, work productively, and contribute meaningfully to community life [66].

This holistic understanding of well-being is critical to our research practices, as it underscores the importance of creating an environment that supports the mental, emotional, and social prosperity of all individuals and the broader community. These principles intersect with the justice-oriented participatory methods that underpin our research, guiding us toward ethical and equitable engagement. The practice of reciprocity and mutuality reflects an ongoing, ethical exchange to foster equality among all team members [12, 36].

In the formative stages of our collaboration, we recognized the integral role of trauma-informed practices as foundational to ensuring psychological safety within the HEARTS Study. Our interpretation of trauma-informed practices is not static; rather, it embodies a dynamic and context-sensitive approach that we have woven into the fabric of our work. We have taken our initial directions from the six principles delineated by the Substance Abuse and Mental Health Services Administration [59]. The principle of safety calls for establishing a secure environment that mitigates risk and fosters each individual's sense of agency.

Secondly, trustworthiness and transparency demand that our strategies and decision-making processes are not only visible but also articulated clearly to maintain relational trust. Thirdly, peer support emphasizes cultivating voluntary, mutual, and respectful relationships, which are paramount to our egalitarian ethos. Fourthly, we aim to redress power imbalances through collaboration and mutuality, ensuring that decision-making is a shared responsibility. The fifth principle, empowerment, voice, and choice, is about recognizing the unique strengths of each team member and creating opportunities for those strengths to be utilized and valued. Lastly, an awareness of cultural, historical, and gender issues obliges us to consciously avoid perpetuating stereotypes and to address historical traumas with humility and inclusivity [47].

From the perspective of the PARES, reflecting on our journey so far, we recognize that the initial stages of engagement facilitated by the GS challenged many of our preconceived notions of hierarchical academic processes; it was not an interrogation but an invitation to an open dialogue. This approach was both disarming and empowering, allowing us to voice our assumptions, biases, and concerns candidly. It was a departure from traditional academic encounters, where many of us have felt subjected to scrutiny rather than being embraced as equal partners. This initial experience served as a precursor of the study’s underlying ethos, where the Primus Inter PARES was not just professed but practiced. The space created for many of us was transparent and transformational, signalling that our well-being was not peripheral but central to the research’s success. However, upon critical self-evaluation, we acknowledge that while this engagement model was profoundly impactful for many of us, it also surfaced challenges. The shift from passive followers to active PARES required us to navigate unfamiliar territory, where the lines between contributing and being overwhelmed often blurred. Some of us thrived in this environment of self-determinism, while others grappled with the weight of this newfound agency.

With time and patience bolstering us, we are navigating towards a shared autonomy, a state where leadership is distributed, allowing any of us to step forward *without* the obligation to do so. This evolving sense of team cohesion has shed light on how traditional research paradigms, with their inherent biases, assumptions, and privileges, once limited our ability to consider the art of the possible in POR engagement and participation. Now, as we reshape these responsibilities to align with our collective vision, we are steadily gaining confidence and momentum. We emphasize the value of distinctiveness over uniformity, acknowledging that both PARES and institutional researchers, and indeed our group as a whole, require the latitude to tailor our approach to *our* unique needs, strengths and personhood.

## Conclusion

This manuscript presents a nuanced approach to POR, anchored in the principles of equality and justice, with a particular focus on partnering with young adults with mental health conditions. Central to our methodology is the principle of Primus Inter Pares: First among Equals, which advocates for an inclusive research environment that values and integrates diverse contributions, thereby enriching the research with varied expertise and experiences. As a team, we emphasize the importance of maintaining the democratic and justice-oriented roots of POR, countering the trend of increasing academization to ensure that our research retains its community-centric values. The significance of our work in POR is its potential to render the field more approachable and accessible to both PARES and institutional researchers. The practical application details (Supplemental file – Appendix A), which include an overview of the strategies and techniques implemented to navigate the challenges encountered in our research (Table 1), are also provided.

**Table 1:** Strategic approaches and practical applications to overcoming challenges in patient-oriented research

**Issues**	**Our Approach**	**Description**	**Practical Application**
Intimidation by professional language and communication barriers	**Equitable Dialogue**	Simplify language, tailor communication, and ensure clarity to remove barriers to meaningful engagement	• 1:1 Meetings,
			• Accessible Blog Posts,
			• Regular Feedback,
			• Transparen-SCI Newsletter,
			• Collaborative virtual workspaces
Lack of trust, past negative experiences, tokenistic inclusion	**Trust Architecture**	Build trust through transparency, acknowledge past experiences, and make all contributions visibly impactful	• 1:1 Meetings,
			• Solidarity Statement,
			• Shared Values,
			• Transparent Communication,
			• Capacity Sharing,
			• Ethics Officer
			• Ombudsperson,
			• PARES personalization
Challenges in sustaining engagement and consultation fatigue	**Community Continuum**	Create reciprocal relationships, acknowledge contributions, and offer capacity sharing for long-term engagement	• WAG Assessment Tool,
			• Cross-learning,
			• Sustainable practices,
			• Focus on intrinsic value,
			• Storytellers Magazine,
			• Writers Circles
Exclusion of diverse groups and systemic discrimination	**Unity in Diversity**	Actively include underrepresented groups, create welcoming spaces, and challenge systemic biases	• Solidarity Statement,
			• Purposive Recruitment,
			• Consensus Building,
			• Rotating leadership,
			• Ethics Officer,
			• Community Builder
			• Resource Library,
			• Public and Private Partners
Hierarchical structures in research and lack of equitable participation	**Shared Stewardship**	Create a research culture where every partner is a custodian of the research process, contributing to and shaping the collective journey	• Rotating Roles,
			• Ombudsperson,
			• Community Builder,
			• Training and Capacity Sharing,
			• Mutual Aid Principles,
			• Increased Transparency,
			• Diversified Communication Platforms
Inflexible systemic frameworks, bureaucratic hurdles, technological barriers	**Agile Frameworks**	Introduce flexible protocols and streamline processes for greater accessibility and inclusivity	• Engagement levels,
			• The Collaboratory,
			• Research Workshops,
			• Communication Groups (WhatsApp, Email, Miro, OneDrive),
			• Flexible Financial Compensation
Emphasis on deficit-based models, unequal power dynamics, infantilization in treatment and research	**Co-Creative Strengths**	Use asset-based models, foster co-creation, and recognize the power that partners can utilize when supportive environments are facilitated.	• Community Builder Role,
			• Building Wellness Support Resources,
			• Psychological Safety and Trauma-Informed Capacity Sharing

Our aim is not to prescribe a specific protocol for replication but to offer a detailed and transparent narrative of our experiences and findings. Our objective was to equip other POR researchers with a foothold, thereby fostering a more efficient exchange of knowledge and averting the redundancy of effort. By presenting this information in an open-source format, we aspire to systemic and structural changes that can facilitate the procurement of essential resources, materials, and support for similar research initiatives.

We contend that the highest level of scientific rigour in POR is achieved *through* a steadfast dedication to equality and the humanistic aspects of research. This approach leads to research outcomes that are impactful but also richly valuable, underscoring the inherent worth, dignity, and strengths of each team member, both PARES and institutional researchers alike. This stance is based on the premise that the most profound expertise, experience, and wisdom are cultivated in environments that prioritize well-being, trust and safety. By establishing conditions conducive to thriving, we create optimal spaces for significant research contributions.

### Supplementary Information

**Supplementary material 1.**

## Data Availability

No datasets were generated or analysed during the current study.

## Abbreviations

PARES: Patient Research Partners
POR: Patient-oriented research
HEARTS Study: Helping Enable Access and Remove Barriers To Support for Young Adults with Mental Health-Related Disabilities
GS: Graduate student
CCHS: Canadian Community Health Survey
WAG: Wellness, Aspiration, and Growth
