# Supplementary material for: Primus Inter PARES: First among equals—practical strategies for young adult PAtient RESearch partners (PARES) by young adult PARES
Source: Res Involv Engagem. 2024 May 8;10:45. doi: 10.1186/s40900-024-00576-0 (PMC11077772; doi:10.1186/s40900-024-00576-0)
Supplement: Supplementary file 1 — Supplementary material 1. [file 40900_2024_576_MOESM1_ESM.zip › Supplemental File - Appendix B.docx]

**Supplemental File – Appendix B – Details of Collaborative Manuscript Process**

Manuscript Process

In the continued interest of transparency with the readership, we also share the process of our manuscript-making (Figure 2), as it required a significant investment of time and effort to ensure inclusive and equitable contribution through the adaptive and differential engagement of all PARES. We started with an ethical framework for our collaborative writing, which was developed in partnership with the HEARTS study ethics officer, GS and Co-Supervisors to think through the elements of what warrants authorship and considerations, addressing the complexities of shared authorship and ensure an ethical approach to contribution and credit. The process exemplifies a deep commitment to participatory research principles, reflecting a concerted effort to navigate and understand the lived experiences of young adults with mental health challenges and translating them into academic scholarship.

The approach we undertook was akin to reverse engineering instead of taking academic manuscripts and turning them into plain language summaries; instead, we took our collective process of working with each other, building relationships and culture, reflexive questions that we are all thinking through and deep engagement with the literature and our experiences in mental health to carve something accessible and meaning and then we just turned that into academic language.

The process was marked by a comprehensive series of individual consultations totalling over 180 hours conducted via digital communication platforms, such as Zoom, text message or email. These initial consultations were essential in accommodating the varied experiences and access levels of PARES, some of whom were engaging in research for the first time. Also, this time provided incredible insights to the GS on the experiences and assumptions PARES had regarding their roles based on previous experiences they had. The GS had regular supervisory meetings to draw upon expertise and experience in creating opportunities for deeper engagement and developing initial mechanisms to cultivate more fulsome engagement.

To establish a common ground of understanding of participatory research and POR necessary for authentic manuscript collaboration, an annotated bibliography comprising over 100 articles was compiled by the GS. Many PARES indicated that despite the summaries, it was still overwhelming and many had limited experiences with such tables and had some concern around the use of the Excel format. Recognizing that this extensive compilation had the opposite effect of confidence and comprehension building, the GS subsequently condensed into Word document lay summaries, not by article but by themes and key ideas. This effort aimed to distill complex academic discussions into more accessible formats, thereby democratizing the information and ensuring all PARES could engage with the material far more meaningfully.

The iterative learning and writing process was facilitated through weekly Writers' Circles, where PARES were introduced to the academic publication process—which seemed to many deeply inaccessible, confusing, gate-kept and elitist. Sessions were provided through Zoom and to accommodate the PARES diverse schedules and time zones, two sessions were offered per day. The word document summaries were reviewed, major themes were discussed juxtaposed with the conversations and experiences of PARES, then in subsequent circles, reflexive questions were asked to interrogate and deconstruct the literature and based on social locatedness and positionality to consider the literature through the lens of experience.

PARES expressed feelings of being "thrown to the wolves" in their previous research engagements or having their “wings clipped.” Alternatively some PARES noted that the IR on the study would often “do all the work” for the manuscript and just put PARES names on it if the PARES agreed. The PARES shared that the IRs of the study gave them the impression as if they were doing the PARES a favour, instead, PaRES felt like a pawn or poster-child for the work, and what the IRs really wanted was to do their traditional research, through their traditional mechanisms, and did not want to work hard to figure out real mechanisms to support their actual engagement.

One PARES provided this illustration of their children wanting to ‘help in the kitchen’ and how, in fact, it was so much more work for them because, in addition to having to teach their kids, they would have to clean up and fix all the mess they made also and the process would take so much longer as a result. Through many different analogies and metaphors, PARES provided this sentiment. On the outside it was as though IRs were making it easier on the PARES by writing the manuscript, but rather, IRs were making it easier on themselves. Underscoring the importance of figuring out non-conventional and non-traditional approaches for POR. As one PARES poignantly shared, "I've felt held back in the past, but this is like building a bridge over the research gap." Our manuscript approach also addressed concerns from PARES who received feedback that they have wanted to be involved in other POR-named research projects, but their "CV was insufficient," providing them with this inclusive opportunity that would build tangible opportunities for them to engage in practical research experiences and to build their academic CV.

An online shared document was utilized for the manuscript's development, with all PARES actively contributing through track changes, comments and writing in text. This approach felt most comfortable to PARES as their engagement with technology is fluid, and most were not only technology-literature but had considerable advancement in their literacy. The GS took the lead in organizing the document's structure in line with journal requirements, integrating content from the oral discussions to provide an initial scaffold. The comments section also served as a discussion space, concept clarifications and PARES talking through word choices they did not like and things that they did not resonate within the POR process. The manuscript process also provided an opportunity to deepen relationships and getting to know each other's perspectives further.

This also provided the opportunity for people to engage in the manuscript in any time zone and when their schedules allowed. One PARES remarked, "This is the first time I've seen my words and experiences genuinely shaping knowledge creation." The online document, complemented by the Writers' Circles, provided a forum to clarify and refine the manuscript, with PARES expressing sentiments such as, “I can’t believe I’m going to be able to contribute to an academic manuscript.” However, we do not want to present the process as without opportunities for improvement. PARES shared frustration about not knowing how to contribute to some parts, and some PARES were also familiar with prior researchers just “doing the work” and then signing off, so the act of getting to put their hands on the document was novel and some felt scared to type directly into the text. PARES were also not used to their oral contributions being turned into written text, so they felt badly about accepting contribution support as this was not offered to them previously, and they were worried about being a “freeloader”. Others felt guilt due to other life commitments and health commitments, and life brought them in and out of the process and that they may get kicked off the study. We share these experiences so that others will be able to set the stage and standard culture and process of a shared contribution and collaborative work.

Of note, an early learning is a completely free-for-all approach was unmanageable, specifically as more PARES were writing directly in the collaborative document; the track changes became messy and hard, even from a health and wellness perspective, such as the multiple red lines on the page. The intention was a collaborative space for all, but when “all” got in, the manuscript became unruly, unmanageable and visually difficult to look. PARES and IRs could not figure out who wrote what, and the comments also became quite unmanageable as more and more people began to contribute. To find a balance between the practical limitations of collaboration and the hierarchies of defaulting to the GS lead/direct all the manuscripts, a Rotational Publication Project Manager (RPM) was created. This role would have oversight on the administrative process, provide dates and deadlines for submissions, and clean up the manuscript so that it was accessible and tidy for partners to engage with and in. For this first publication in the HEARTS Study, the GS is the RPM and also due to the timings of many PARES being in exams.
