# Supplementary material for: Primus Inter PARES: First among equals—practical strategies for young adult PAtient RESearch partners (PARES) by young adult PARES
Source: Res Involv Engagem. 2024 May 8;10:45. doi: 10.1186/s40900-024-00576-0 (PMC11077772; doi:10.1186/s40900-024-00576-0)
Supplement: Supplementary file 1 — Supplementary material 1. [file 40900_2024_576_MOESM1_ESM.zip › Supplemental File - Appendix C - Solidarity Statement.docx]

**Supplemental File – Appendix C – Solidary Statement**

**H**elping **E**nable **A**ccess and **R**emove Barriers **T**o **S**upport for Young Adults with Mental Health-Related Disabilities (The HEARTS Study)

**Last Updated**: Sunday, November 26, 2023.

Last Reviewed: January 5, 2024.

<https://www.naturalreaders.com/online/> (if you prefer to listen)

**Solidarity Statement for the HEARTS Study Team**

Research, any kind of research, has the potential to be unsafe. The process may put a strain on emotions, psychological and physical health; it may have the potential to cause distress, exacerbate emotional traumas, stigma in home communities, and strained or fragmented social relationships. Moreover, participatory research may be even more susceptible to these difficulties due to its involvement with individuals who are close to the content of the research. As such, everyone, including ourselves, involved in participatory research is called upon to be proactive in creating a supportive atmosphere where holistic safety, psychological, spiritual, physical, emotional and material safety are ensured and validated.

**Section 1: Introduction to the Solidary Statement for the HEARTS Study**

Working together in the HEARTS research study is a collective voyage dedicated to the exploration and enhancement of our communal well-being. At the core of this Solidarity Statement is the principle of mutual aid (*which is a form of solidarity-based support where communities unite to meet each other's needs, recognizing that the systems in place are inadequate to do so and often exacerbate disparities*). It's a practice grounded in reciprocal care, collective empowerment, and the recognition that addressing individuals' immediate needs also requires challenging the conditions that create inequity.

This serves as a waypoint in our working together to build emancipatory (*which means liberation from any form of social, political, or economic restrictions or constraints. It embodies the actions and principles aimed at achieving freedom and equality, often in the context of uplifting oppressed or minoritized groups*) knowledge. This Solidarity Statement stands as our declaration of unity and commitment, shaping how we interact with one another and affirming our dedication to the communities we are here to understand and support.

**Shared Journey of Inquiry and Growth**

The HEARTS study transcends the boundaries of conventional or “traditional” hierarchical research. It is a collaborative journey, embodying our interdependence and the power of collective knowledge, ethics, compassion, and support. We unite as coresearchers driven by a common purpose to conduct research that is not only insightful but catalytic in fostering transformative change.

**Our Values and Vision**

At the heart of our study are the values of emancipation, solidarity, respect, dignity, diversity and community. We recognize that the well-being of each individual is inseparably linked to the collective health of our society. Our research practices are built on a foundation of solidarity, ensuring that each coresearcher and coresearcher in the study is acknowledged, heard, and valued. We respect the diverse contributions and lived and living experiences of all, creating space to actively engage in a process that honours their agency, autonomy, self-determination and insights. We seek to deepen our understanding and improve the collective well-being through our collaborative efforts.

**Invitation to Collaborative Participation**

This Solidarity Statement invites you to engage with the HEARTS study actively and openly. It is a dynamic manifesto that adapts to the evolving nature of our work and the changing contexts we navigate. We encourage you to bring your whole self to this process, sharing your unique insights, experiences, and expertise to enhance our collective understanding.

**Respecting Individual and Collective Journeys**

We acknowledge the personal narratives and challenges that intersect with our collective path. The HEARTS study is committed to fostering a safe, inclusive, and nurturing environment. Recognizing that life is unpredictable, we offer flexibility in participation and support each individual's journey with understanding and compassion.

**Mutual Aid: Our Methodology in Action**

Informed by the principles of mutual aid, our research emphasizes cooperation, reciprocity, and community-driven action. Mutual aid is not merely a theoretical concept; it is the practice of providing support based on mutual respect and shared understanding. It is about working together, pooling our resources and knowledge, and learning from one another for a common purpose—that is bigger than ourselves.

**Challenging Systems of Oppression**

We are keenly aware that systemic oppression has long dictated whose voices are amplified and whose are silenced. We strive to dismantle barriers of ableism, racism, sexism, classism, and other discriminatory practices by creating spaces that are inclusive and just. Our approach celebrates diversity and amplifies every voice, ensuring that our collective work is rich with varied experiences and perspectives.

**Inclusivity and Justice in Practice**

Our research space reflects our commitment to inclusivity and justice. We engage in practices that are equitable and honour each coresearcher as a vital member of our shared endeavour. We believe in the power of participatory democracy, where every coresearcher is an essential contributor to the research.

Inclusive Summary:

The HEARTS study is a team effort to enhance community well-being, guided by "mutual aid"—the idea of people supporting each other when systems fall short. Our Solidarity Statement is a commitment to work together with respect and value everyone's contributions.

We invite you to participate in a way that fits your life, knowing that flexibility and support are key. We emphasize teamwork, sharing, and learning from each other, aiming to overcome societal barriers and ensure diverse voices shape our study.

In essence, we're more than researchers; we're a community striving for fairness and inclusion, where every voice is crucial to our shared purpose.

**Section 2: Purpose and Scope**

The purpose of this Solidarity Statement is to articulate the foundational principles that will guide our collective work in the HEARTS study. This document is not merely a set of guidelines but a living testament to our commitment to meaningful participatory research. It is designed to underpin every interaction and decision, ensuring that our research is not only effective but also ethical, inclusive, and transformative.

**Understanding Mutual Aid in Research**

Mutual aid, as a principle, involves the reciprocal exchange of resources and services for mutual benefit. In the context of participatory research, mutual aid rejects traditional hierarchical models and instead centers on collaboration and shared power. It sees young adults not as commodities or subjects but as coresearchers with valuable knowledge and experiences that are essential to the integrity and success of the study.

**Defining Our Collective Approach**

The HEARTS study is operationalized through practices that prioritize the well-being of all involved, recognizing that each coresearcher brings their whole self to the research. This approach is inherently anti-oppressive, actively dismantling power structures that marginalize or silence certain voices. We strive to create spaces where individuals feel valued, heard, and respected, fostering a sense of community and shared purpose.

**Scope of the Solidarity Statement**

This statement applies to all facets of the HEARTS study—from initial planning and data collection to analysis and dissemination of findings. It frames our interactions, shapes our meeting structures, and guides our resolution processes. It is relevant to every member of the research community, whether they engage daily or sporadically, in public or private capacities.

**Mutual Aid as an Ethical Compass**

The mutual aid principles within this Solidarity Statement serve as our ethical compass. They remind us that our work is not merely about achieving research outcomes but about how we reach those outcomes—through processes that embody the values of equity, justice, and solidarity. Our research methods are as important as our findings, for they reflect our commitment to social change.

**Dynamic and Evolving Nature**

The Solidarity Statement is dynamic, acknowledging that as our study evolves, so too will our understanding. We commit to regularly revisiting this document, incorporating feedback, and adapting our practices to ensure they remain aligned with our collective values and the needs of our community.

Top of Form

Inclusive Summary:

Our Solidarity Statement lays out the core values guiding our HEARTS study, ensuring our work is ethical, inclusive, and impactful. We embrace 'mutual aid'—equal collaboration and shared knowledge—for a research model that is fair and beneficial to all participants.

This document is a commitment to anti-oppressive research, where everyone's voice matters and is respected. It applies to all stages of the study and to all members, whether their involvement is big or small, public or private.

As our study progresses, we'll keep updating this statement to stay true to our values and respond to our community's needs. We're committed to research that not only seeks answers but also stands for equity, justice, and solidarity.

Bottom of Form

**Section 3: Consent and Participation**

**Understanding Consent in Participatory Research**

Consent, within the context of participatory research, is the foundational pillar that upholds the dignity and autonomy of every individual involved in the HEARTS study. It is a dynamic process that goes beyond the mere act of agreeing to participate; it involves a continuous conversation about each person’s involvement and comfort levels throughout the research journey.

**The Core Principles of Consent**

In the HEARTS study, we adhere to the following core principles to ensure that consent is informed, ongoing, and rooted in mutual aid values:

- **Informed**: Co-researchers are provided with comprehensive information about the study's aims, methods, potential impacts, and their expected role. This transparency allows coresearchers to make educated decisions about their involvement.
- **Voluntary**: Participation is a voluntary act. Co-researchers have the right to partake in the study out of their own free will and aligned with their personal values and capacities.
- **Revocable**: Consent is not a binding contract but a revocable agreement. Coresearchers have the right to withdraw their consent and discontinue their involvement at any stage of the research without penalty or loss of benefits.

**The Process of Obtaining Consent**

Consent is not a one-off event but a process that occurs at multiple stages:

- **Initial Consent**: Before participation, individuals are engaged in a dialogue that covers all aspects of the study. Questions are encouraged; clear, jargon-free language is used to facilitate understanding.
- **Ongoing Check-ins**: Regular check-ins are conducted to reaffirm each individual’s continued consent. These check-ins are essential to accommodate any changes in the research or the coresearchers’ circumstances.
- **Documentation**: While there is no formal consent, unlike research participants, we do value verbal affirmations and non-verbal cues as part of the consent process, ensuring it is accessible to all, regardless of literacy or disability.

**Navigating Participation Barriers**

We acknowledge that barriers—be they linguistic, cultural, technological, or personal—can impede the ability to participate fully in the research process. We are committed to an approach that includes:

- **Personalized Support**: Offering one-on-one support sessions to discuss participation in a safe and confidential environment.
- **Flexible Engagement**: Recognizing that life’s circumstances can affect participation, we offer various ways for individuals to engage with the study, respecting personal limits and capacities.
- **Cultural Sensitivity**: We strive to understand and respect cultural differences that might influence engagement, ensuring our practices are centred on respect and inclusive of diverse backgrounds.

**You’ve got the Power - Coresearchers**

Mutual aid principles guide us to recognize the power that each coresearchers has and the power in the relations between us. As such, the HEARTS study encourages:

- **Active Engagement**: Coresearchers are invited to contribute to the research actively, bringing their unique insights and expertise to the study.
- **Shared Decision-Making**: Co-researchers are involved in making decisions about the study design, data interpretation, and dissemination of findings.
- **Capacity Sharing**: Through workshops and training, we aim to enhance all coresearchers' (this means all individuals) skills and knowledge, enabling every person to contribute more effectively to the research and their communities.

Inclusive Summary:

In the HEARTS study, consent is about making sure everyone involved feels comfortable and informed every step of the way. It's not just about saying “yes” once but having ongoing conversations to make sure everyone is still on board. We make sure participants know exactly what the study involves and let them decide if they want to be a part of it without any pressure. If they change their mind, they can leave at any time.

We also understand that not everyone can participate in the same way, so we offer support and flexibility. We're sensitive to cultural differences and want to ensure no one isleft out. Our study is all about working together, making decisions as a team, and helping each participant grow by learning new skills. This is how we do research with respect, dignity, community and compassion.

**Section 4: Power Dynamics**

Power dynamics are present in all social interactions and can deeply influence the outcomes of our collaborative work, especially in research contexts. In the HEARTS study, we recognize the layers of power that exist due to social hierarchies, institutional roles, and personal experiences. This awareness is crucial as we aim to create an equal (*not that everything is the same, but that all people are equal in their dignity and worth*) and equitable research environment that truly values the voices and contributions of all co-researchers.

Power dynamics can manifest in various ways, often subtly. They can influence who feels able to speak or contribute, whose ideas are taken seriously, and who assumes leadership or decision-making roles. In a participatory research context like ours, failing to address these dynamics can replicate the very systems of oppression we seek to understand and dismantle.

To mitigate these dynamics, we commit to the following practices:

1. **Equitable Participation**
   - We will establish mechanisms that ensure all co-researchers have equal opportunities to engage in the research process. This includes creating spaces where individuals from diverse backgrounds can contribute without the fear of their input being undervalued. This includes addressing historical and ongoing, structural and systemic effects of minoritization, oppression and injustice where it applies to this research study.
2. **Shared Leadership**
   - Leadership roles will rotate among co-researchers to distribute power and prevent hierarchical structures from forming. This practice encourages a variety of perspectives to guide the research direction and acknowledges the value of shared expertise.
3. **Transparent Decision-Making**
   - Decision-making processes will be transparent, with clear documentation and open communication channels. This transparency ensures that all co-researchers understand how decisions are made and have the opportunity to contribute to those decisions.
4. **Ongoing Self-Reflection**
   - We will engage in regular self-reflection and group discussions to examine how power dynamics are playing out within our research team. This includes acknowledging and addressing any behaviours or structures that may perpetuate inequality.
5. **Capacity Sharing**
   - We will provide training and resources to all co-researchers, especially those who may feel less confident in a research setting due to systemic barriers or personal experiences. This may include workshops on research methods, ethics in research, and presentation skills.
6. **Inclusive Language and Practices**
   - Our communication will be conscious of the power inherent in language. We will use inclusive, non-jargonistic language that respects and reflects the diverse backgrounds of our co-researchers.
7. **Conflict as Opportunity for Growth**
   - We view conflict not as a failure, but as an opportunity for growth and deeper understanding. We will approach conflicts with a transformative justice perspective, seeking to address the root causes and learn from these experiences.
8. **Responsive and Adaptive Approach**
   - We will remain flexible and responsive, adapting our practices as we learn from our experiences and from the feedback of co-researchers. This includes being open to changing power dynamics that are not serving the group's goals.

By embracing these principles, we aim to not only study but also embody mutual aid within our research process. Mutual aid, at its core, is about building networks of care that resist oppressive systems. It is about recognizing that we are interdependent and that our well-being is bound up with the well-being of others. In the HEARTS study, we are committed to an approach that is deeply democratic, participatory, and transformative, enabling all co-researchers to thrive as valued members of our collective endeavour.

Inclusive Summary:

The HEARTS study is committed to ensuring that everyone has an equal chance to contribute and make decisions. We're intentional about power dynamics to make sure no one's voice is overlooked, and we rotate leadership roles to share power fairly. All decisions are made openly so everyone can see how and why choices are made. Regular check-ins help us stay aware of any power imbalances, and we offer training to make sure everyone can participate fully, regardless of their background.

We talk openly, using language everyone can understand, and see conflict as a chance to learn and improve. Our study embraces mutual aid values, recognizing that we all rely on each other, and by working together democratically, we all grow stronger.

**Section 5: Emotional Labour and Well-being**

**Understanding Emotional Labour in Participatory Research**

The concept of emotional labour refers to the process by which individuals manage and regulate their emotions to align with the expectations of an organization or as a response to the situational demands of a role. In the context of participatory research, emotional labour encompasses the efforts of co-researchers to navigate their own emotional responses while engaging with potentially sensitive or triggering topics, as well as the work of maintaining a supportive and constructive atmosphere within the research collective. Emotional labour in participatory research is particularly pertinent because co-researchers often engage with subject matter that is closely tied to personal experiences, community struggles, or social injustices. This deep connection can evoke strong emotional reactions, ranging from passion and solidarity to grief and frustration. The intensity of these emotions is a testament to the co-researchers' commitment to the subject matter, but it also necessitates a conscientious approach to emotional well-being.

**Commitment to Emotional Well-being and Psychological Safety**

In recognition of the substantial emotional labour that co-researchers contribute to the HEARTS study, we are steadfast in our commitment to fostering an environment where emotional well-being is prioritized. This commitment is multifaceted and includes:

1. **Creating Safe Spaces**: We will cultivate spaces—both virtual and, where applicable, physical—that are safe for emotional expression and vulnerability. These spaces will allow co-researchers to share their feelings, experiences, and insights without fear of dismissal or reprisal.
2. **Facilitating Emotional Support**: Acknowledging the potential for research topics to resonate deeply with personal experiences, we will facilitate access to emotional support services. This may include providing referrals to community services, establishing peer support networks, and integrating mental health breaks into our research schedule.
3. **Encouraging Self-Care**: We will encourage co-researchers to engage in self-care practices that sustain their emotional, spiritual and mental health. This encouragement is not merely rhetorical; we will actively incorporate self-care reminders into our gatherings/meetings/labs/workshops and communications, and we will respect the needs of co-researchers to step back or take time off when necessary for their well-being.
4. **Building Capacity in Trauma-Informed Practices**: Our research team will build capacity in trauma-informed practices to ensure that we are equipped to respond to the emotional needs that may arise during the research process. This will also help us recognize the signs of emotional distress and address them promptly and compassionately. This will be an ongoing conversation to understand issues of safety, choice, autonomy and collectivism, wellness and to understand what is within the scope of this research study.
5. **Promoting Collective Care**: Beyond individual self-care, we recognize the power of collective care—supporting each other as a community of co-researchers. We will promote practices of collective care, such as regular check-ins, shared reflection sessions, and the acknowledgment of the emotional labour that each co-researcher contributes.
6. **Valuing Emotional Contributions**: The emotional insights that co-researchers bring to the study are invaluable. We will honour these contributions and the vulnerability, courage and bravery that accompanies them by ensuring they inform our research findings and recommendations.

**Note**: The emotional labour inherent in participatory research is not a burden to be shunned but an integral aspect of the work that deserves recognition and support. Through our commitment to emotional well-being, we affirm the importance of each co-researcher's emotional journey and its impact on the collective research endeavour. In doing so, we fortify the foundation of our solidarity and enhance the depth and authenticity of our collaborative inquiry.

Inclusive Summary:

The HEARTS study acknowledges the emotional effort that comes with research. We're dedicated to looking after the emotional well-being of all team members. Here's how we do it:

**Safe Spaces**: We've created welcoming spaces for open and honest conversation about feelings and experiences.

**Emotional Support**: We're ready to help connect you with support services and peer networks, recognizing that our research might touch on personal and sensitive areas.

**Self-Care**: We remind each other to take care of ourselves and respect everyone's need for breaks.

**Learning Together**: We're learning about trauma-informed practices to better support each other, especially when someone's having a tough time.

**Caring as a Team**: We look out for each other, with regular check-ins and group sessions to share what we're going through.

**Valuing Emotions**: Your emotional input is a big deal to us. It helps make our research real and relatable.

Emotional labour is part of our journey, and we take it seriously, ensuring it shapes our study in meaningful ways and not harmful or harming ways.

**Section 6: Communication and Virtual Etiquette**

Communication is the lifeblood of any collaborative effort, especially in the context of participatory research, where diverse voices converge to contribute to a collective goal. The efficacy of our communication shapes the quality of our interactions and the outcomes of our research. In a virtual environment, where non-verbal cues are often absent, the clarity, respectfulness, and intentionality of our words gain even more significance.

**Virtual Etiquette and Considerations:**

In the digital realm of the HEARTS study, we uphold virtual etiquette not merely as a set of rules but as a manifestation of our mutual respect and commitment to inclusive dialogue. These guidelines are crafted to ensure that every coresearcher feels heard, valued, respected and able to meaningfully contribute:

- **Active Listening:** Engage in active listening by giving full attention to the speaker, acknowledging their contributions, and avoiding interrupting or speaking over others. Active listening also includes reading carefully when others communicate in writing and responding thoughtfully.
- **Turn-taking:** Utilize features like 'raise hand' in video calls or appropriate pauses in conversation to signal your desire to speak, ensuring that all coresearchers have the opportunity to contribute without the need for over talking or interruptions. Consider the three-before-me method—which is allowing three other coresearchers to speak before speaking again; or the Step Up/Step back method—which is a practice implemented in meetings and training to balance participation, encouraging individuals who frequently speak to listen more, and those who are usually quiet to contribute. This approach not only addresses the dynamics between introverts and extroverts, but also promotes inclusivity by urging those from historically dominant groups to create space for historically/structurally and systematically minoritized identities to lead and express themselves. Coresearchers that do not feel comfortable speaking should have space to engage how they feel comfortable, whether through the chat function, private messaging, or orally if they choose.
- **Mute Courtesy:** Keep your microphone muted when not speaking to minimize background noise and distractions, thereby maintaining the focus and clarity of the conversation.
- **Respectful Address:** When addressing each other, use the names and pronouns that each coresearcher identifies with, as this is a fundamental expression of respect for individual identity and dignity.
- **Trigger Warnings and Mindful Sharing**: When speaking about personal experiences, we commit to using proactive statements that precedes potentially distressing or traumatizing content, providing an opportunity for individuals to prepare or disengage. We will also engage in conscious and intentional communication of information or experiences in a manner that minimizes emotional harm to others, considering both the content and context.

**Constructive Dialogue:**

Constructive dialogue is the cornerstone of meaningful participation and decision-making. It involves:

- **Clarity:** Be clear and concise in your communication to avoid misunderstandings. Use common language over jargon to ensure accessibility to all, regardless of their academic or professional background.
- **Constructive Feedback:** Offer feedback that is specific, actionable, and delivered with respect and kindness. The goal is to build, not break, and to improve, not impair our collective work.
- **Conflict Engagement:** Recognize that disagreements are a natural part of collaborative work. We want the diversity of ideas, experiences and points of view. Approach conflicts with a mindset geared toward resolution and understanding, rather than contention, defensiveness or avoidance.

**Confidentiality and Privacy:**

- **Sensitive Information:** Treat all shared personal stories, data, and insights with the utmost confidentiality. Do not record or disseminate any part of our discussions without explicit consent from all parties involved.
- **Data Security:** Adhere to best practices in data security to protect the information and privacy of our coresearchers. This includes using secure platforms for communication and data storage.

**Regular Review and Adaptation:**

- **Feedback Mechanism:** Establish a regular feedback mechanism to discuss the effectiveness of our communication practices and to adapt as needed. This ensures that our approach remains dynamic and responsive to the group's needs.

Inclusive Summary:

In the HEARTS study, we know that good communication is key, especially online where we can't rely on body language. Here's how we keep things clear and respectful:

**Active Listening**: We really listen to each other, waiting our turn to talk and keeping our mics muted when we're not speaking.

**Speak Clearly**: We say what we mean without jargon, making sure everyone can follow along.

**Be Kind**: We're all about building each other up, so when we give feedback, it's done gently and with the intent to help.

**Handle Disagreements**: We're okay with not always agreeing, but we handle our differences with the aim of understanding, not arguing.

**Keep It Private**: What's shared in our study stays in our study. We're serious about keeping personal info and discussions confidential.

**Stay Secure**: We use safe, secure ways to chat and share data, protecting everyone involved.

**Always Improving**: We regularly check in to see how we can communicate better, making changes where needed.

**Section 7: Anti-Oppression Commitment**

**Understanding Anti-Oppression**

Anti-oppression is a framework and a commitment that seeks to recognize, address, and dismantle the structural, systemic and interpersonal dynamics that minoritize individuals and groups while privileging others. It is an active and ongoing process of shifting power dynamics toward equity and justice within our research collective and the broader community.

**Our Commitment**

In the HEARTS study, we acknowledge the pervasive nature of systemic injustices, including racism, colonialism, sexism, ableism, classism, homophobia, transphobia, and other forms of discrimination. We recognize that these injustices are not isolated or individual occurrences but are embedded in the very structures of our society. Therefore, we are committed to actively challenging these systems within the space of our research.

**Implementing Anti-Oppression**

- **Continuous Learning**: We will engage in ongoing education about power, privilege, and oppression, including how these dynamics manifest in research practices. We will provide resources for all co-researchers to deepen our collective understanding and to develop practical skills to combat oppressive dynamics.
- **Inclusive Practices**: We will employ methodologies that honour the lived experiences of minoritized/equity-deserving groups and prioritize their voices in the research process. This includes ensuring that research questions, methods, and analyses are informed by those most impacted by the issues we study.
- **Equitable Participation**: We will strive to create spaces where all co-researchers can participate fully and authentically. We will work to eliminate barriers to participation, whether they be language, accessibility, technology, or other factors that may prevent full engagement.
- **Reflection and Critique**: We encourage co-researchers to critically examine their own positions of power and privilege and to understand how their actions and words may perpetuate oppression. We promote a culture where self-reflection is valued and where we hold each other accountable with compassion and understanding.
- **Community Accountability**: We are dedicated to creating processes that allow for community-driven accountability, where the group collectively addresses instances of oppression and works towards transformative solutions.
- **Structural Analysis**: We will examine how institutional policies and practices may reinforce inequities and work to propose research-driven changes that advocate for structural transformation.

**Cultivating Safe Spaces**

- **Active Allyship**: We will support co-researchers in developing the skills to be effective allies/accomplices to minoritized/equity-deserving groups, actively resisting oppressive behaviors, and supporting those who are targeted by discrimination.
- **Conflict Transformation**: We understand that addressing oppression can lead to conflict. We are committed to transforming conflicts through processes that are restorative and that promote healing and growth.
- **Trauma-Informed Approach**: We acknowledge that discussing and confronting oppression can be traumatic. Our approach will be sensitive to the trauma that can be associated with these experiences, ensuring that we prioritize the emotional well-being of co-researchers.

**Beyond the Research**

- **Solidarity in Action**: We commit to extending our anti-oppression efforts beyond the confines of our study, supporting community initiatives that align with our values and using our research findings to advocate for systemic change.
- **Feedback and Evolution**: We will regularly solicit feedback on our anti-oppression efforts and will be open to evolving our practices in response to new insights and the changing needs of our community.

In embracing this anti-oppression commitment, we recognize that this is not a checklist or a one-time task but a continuous journey toward creating a just and equitable world. Through our research, we aim to contribute to this transformative process, both within our collective and in the wider society.

Inclusive Summary:

In the HEARTS study, we're all about knocking down the walls of oppression—things like racism, sexism, and all those other 'isms' that unfairly keep some people down while lifting others up. Here's our plan:

Learn and Grow: We're on a never-ending journey to understand more about power and privilege, and we're sharing this learning so we can all get better at fighting oppression.

Everyone's Voice Matters: We're making sure that the voices of people who often get ignored are front and center in our work.

No Barriers: We're tearing down the obstacles that get in the way of everyone being fully involved, whether that's language, tech issues, or something else.

Looking in the Mirror: We're encouraging everyone to think about their own power and how to use it for good, not to keep others down.

Work Together: We're all about community accountability—working as a team to call out and fix oppressive behaviours and practices when we see them.

Safe Spaces: We're committed to being there for each other, helping everyone to be a good ally and to work through conflicts in ways that heal, not harm.

Beyond the Study: Our fight against oppression doesn't stop with the research; we're using what we learn to help make the whole world a fairer place.

By sticking to these points, we're not just doing research—we're building a mini-society that's fair, kind, and just for everyone involved.

**Section 8: Conflict Resolution Framework**

Conflict is a natural part of human interaction, especially when engaging in collective efforts like participatory research. It is not the presence of conflict that challenges the integrity of a group, but rather the absence of a thoughtful approach to understanding, managing, and resolving it.

Our approach to conflict resolution is not punitive but restorative. It is rooted in the understanding that each person's voice is valuable, and that disagreement can be a powerful tool for growth and learning. By addressing conflicts through a lens of compassion and mutual support, we aim to not only resolve issues but to strengthen our community and enhance our collective work.

**Principles**

1. **Collective Care**: Recognizing that individual well-being is interlinked with the well-being of the group, our conflict resolution process prioritizes the emotional and psychological safety of all members.
2. **Transparency**: All steps taken in the conflict resolution process will be transparent to ensure trust remains intact among all coresearchers. There process will be transparent, however the names of individuals may be held in confidence/privacy as determined by the parties involved.
3. **Active Listening**: Conflicts are opportunities to practice active listening, seeking to understand diverse perspectives and experiences fully.
4. **Non-Violence**: Our approach is grounded in non-violence, aiming for dialogue and understanding over coercion or force.
5. **Equity**: We strive to give equal voice to all parties involved, particularly uplifting those who may be minoritized or less heard.
6. **Confidentiality & Privacy**: Sensitive issues are addressed with the utmost respect for the privacy and dignity of those involved.

**Process**

1. **Direct Communication**: We encourage direct, but respectful, communication where possible, to address the heart of the conflict swiftly, constructively and compassionately.
2. **Identification and Acknowledgment**: When a conflict arises, it is promptly and openly acknowledged by the group, ensuring that it is neither ignored nor allowed to fester.
3. **Safe Space for Dialogue**: We create a safe space where parties can discuss the conflict without fear of retribution or judgment.
4. **Understanding the Conflict**: Efforts are made to understand the root causes and the perspectives of all parties. This involves looking beyond immediate tensions to broader systemic issues that may be contributing to the conflict.
5. **Peer Support**: Coresearchers will be matched in buddies to be able to receive support, mentorship and coaching to support the resolving of conflicts and the balancing of power.
6. **Anonymous Reporting**: Recognizing the need for safety in voicing concerns, we offer channels for anonymous reporting where necessary.
7. **HEARTS Ombudsperson**: For issues that involve the PI/co-researcher of the study, there will be an arms-length Ombudsperson that co-researchers can access to help resolve conflicts and concerns that co-researchers do not feel safe or comfortableadressing directly.
8. **Seeking Solutions Collectively**: Solutions are sought through consensus, with all parties contributing to a resolution that meets everyone's needs and upholds the study's values.
9. **Implementing Agreements**: Agreements reached are clearly articulated, implemented with the consent of all involved, and followed up to ensure they are effective.
10. **Learning and Adapting**: Post-resolution, the group reflects on the conflict and resolution process to glean lessons that can inform future practices, preventing similar issues and strengthening the group's resilience.

**Reintegration and Healing**

Post-conflict, we focus on healing and reintegration, ensuring that individuals who were in conflict can continue to engage in the research process productively and feel supported by the entire team.

**Commitment to Collective Care**

Above all, our accountability mechanisms are imbued with our commitment to collective care. We understand that the well-being of each individual is integral to the well-being of the whole. We strive to create a space where each person feels seen, heard, and valued, and where accountability is not a tool for discipline but a means for communal healing and growth.

In aligning with the principles of mutual aid, this accountability framework within the HEARTS study ensures that we move forward together, not just as co-researchers, but as a community deeply invested in the transformative power of solidarity and mutual respect.

Inclusive Summary:

In our HEARTS study, when disagreements or misunderstandings pop up, we handle them with care, not blame. Here's how we do it:

We Look After Each Other: If there's a problem, we tackle it with everyone's feelings in mind, making sure we all feel safe and heard.

Clear and Open: We're like an open book about how we sort out issues, so everyone knows what's going on and can trust the process.

Listening is Key: We really listen to each other, trying to understand all sides of the story.

No Fighting: We're all about talking it out—no shouting matches or arm-twisting.

Everyone's Equal: No matter who you are, your voice matters just as much as anyone else's when we're solving problems.

Private Stuff Stays Private: We're super careful with personal info and delicate matters.

If we hit a snag:

We talk to each other directly, but always with kindness.

We get what's bugging us out in the open ASAP.

We make a safe space to chat without anyone feeling on edge.

We dig deep to figure out the real reasons behind the conflict.

We stand by each other, offering help and advice to work through the tough bits.

If you're not comfy saying it out loud, we've got a way to report issues quietly.

If the problem is with the big boss, we've got an impartial helper to sort it out.

We all chip in to find a fix that works for everyone.

We make sure everyone's cool with the fix and check it's actually working.

Afterward, we all think about how it went and what we can learn to avoid the same hassle next time.

And once the air's cleared, we focus on getting back to being a team, making sure everyone's feeling okay and ready to dive back in. Because at the end of the day, we're more than just a bunch of researchers—we're a community this is looking to make things better for everyone.

**Section 9: Exit and Re-engagement Protocols**

As an integral part of our commitment to mutual aid and solidarity within the HEARTS study, we acknowledge the dynamic and sometimes unpredictable nature of life. Coresearchers in this research journey may encounter personal, professional, or health-related circumstances that necessitate stepping back from the study. In alignment with our principles, we emphasize that participation in the HEARTS study is voluntary and based on active, ongoing consent. The well-being of each co-researcher is paramount, and we understand that one's ability to contribute may change over time.

**Exiting the Study**

Should you find yourself needing to withdraw from the study, we ask that you:

- **Notify the Team**: Communicate your need to step back to the research team. This notification does not need to include personal details but should provide sufficient notice to allow for any transitions necessary for the continuity of the research.
- **Transition of Responsibilities**: If you have specific duties or have undertaken certain tasks, we encourage a discussion about how these can be transitioned to other team members or put on hold, ensuring no undue burden falls on other coresearchers.
- **Debriefing**: If comfortable, engage in a brief feedback session. This is an opportunity to share any insights that could improve the experience for existing and future co-researchers.
- **Confidentiality**: Upon exiting, maintain confidentiality regarding any sensitive information related to the study, respecting the privacy and integrity of the work, and its coresearchers.

**Re-engagement in the Study**

We recognize that departure from the study need not be permanent. Life circumstances change, and the door remains open for past coresearchers to return. Re-engagement is welcomed and will be facilitated by:

- **Reorientation**: A refresher on any updates or changes that have occurred during your absence to ensure you can integrate smoothly back into the study.
- **Flexible Engagement**: Offering a variety of roles and levels of involvement that can accommodate your current capacity and interests.
- **New Consent**: Ensuring that your return is marked by a renewed understanding and agreement to the study's terms, as these may have evolved since your initial consent.

**Support Throughout**

By fostering an environment where coresearchers feel they can openly communicate their needs and changes in their capacity to engage, we honour the core tenets of mutual aid—solidarity, support, and communal care. This respectful acknowledgment of each person's evolving situation is foundational to building trust and a thriving research community.

Top of Form

Inclusive Summary:

Life's full of surprises, and sometimes, coresearchers in our HEARTS study need to hit pause or step away for a bit. Here's the simple scoop on how we handle those moments:

Taking a Break:

Just let us know: Give us a heads up if you need to take a break.

Handing over the baton: If you've got tasks on your plate, we'll figure out together how to pass them on.

Let's chat before you go: If you're up for it, a quick chat to share any thoughts on making the study even better would be awesome.

Please keep the secret stuff secret, even after you've stepped back.

Thinking of Coming Back?

Catch-up: We'll fill you in on anything new so you're up to speed.

Choose your own adventure: We've got all sorts of ways you can jump back in, depending on what's good for you.

Yes, let's do this (again): We'll just double-check you're cool with how things work now, as they might have changed since last time.

Always Here for You:

This whole come-and-go policy is about making sure everyone feels comfy and respected. It's about staying true to our vibe of sticking together and looking out for each other, no matter what life throws our way.

Bottom of Form

**10. Safety and Security Considerations**

As an integral part of our commitment to mutual aid principles within the HEARTS study, we prioritize the safety and security of all coresearchers involved. This section delineates our approach to ensuring that every individual feels secure and protected throughout the research process.

**Personal Safety Measures:**

- We emphasize the importance of creating a secure environment for all co-researchers. This involves establishing clear protocols that prioritize personal safety, particularly when engaging in fieldwork or community interactions.
- We encourage co-researchers to share their safety concerns and experiences so that the research team can address them promptly and effectively.

**Data Security and Confidentiality:**

- Protecting the personal data and information of our coresearchers and study participants is paramount. We adhere to stringent data protection regulations to ensure that all research data is securely stored and accessed only by authorized personnel.
- Confidentiality agreements will be in place to reinforce our commitment to non-disclosure of sensitive information.

**Psychological Safety:**

- Recognizing that participation in research can sometimes involve emotional labour, we strive to create spaces where co-researchers can express themselves without fear of judgment or reprisal.
- Support systems, such as peer networks and access to community services, will be provided to help manage any psychological stress or trauma that may arise during the study.
- A Community Builder role has been created to develop the support mechanisms.

**Digital Security:**

- With the increasing importance of virtual communication and data sharing, we will implement robust digital security measures to safeguard against unauthorized access and cyber threats.

**Reporting and Response Procedures:**

- A clear and accessible reporting mechanism will be established for any safety or security concerns. This will allow for timely and appropriate responses to any incidents that may occur.
- We will maintain an open line of communication for safety-related issues, ensuring that coresearchers can easily reach out for support when needed.

**Creating a Culture of Safety:**

- Above all, we endeavour to foster a culture where safety and security are everyone's responsibility. By encouraging vigilance, mutual support, and collective care, we aim to create a research environment where all coresearchers can work together in a safe and secure manner.

Inclusive Summary:

Personal Safety: We're committed to a safe environment and encourage discussing any safety concerns.

Data Protection: Strict measures ensure your personal information stays secure and confidential.

Emotional Support: Spaces for open expression and resources for emotional support are provided.

Digital Safeguards: We implement strong digital security to protect our online interactions and data.

Incident Reporting: Easy-to-use reporting systems are in place for swift support and resolution of safety issues.

Safety as a Shared Value: A collective commitment to watch out for one another's safety and well-being.

**Section 11. Inclusion of Diverse Voices**

In our commitment to the principles of mutual aid and participatory research, the HEARTS study is deeply invested in the inclusion of diverse voices, particularly those that have been historically minoritized or silenced. The inclusion of these voices is not merely a gesture towards diversity but is a fundamental aspect of ethical research practice and a core element of our collective commitment to social justice and transformative action.

**Understanding the Value of Diverse Voices**

Mutual aid, at its core, is about collaboration and support among community members, recognizing that all individuals bring unique perspectives and experiences that are invaluable to the collective wisdom of the group. The inclusion of diverse voices ensures that the research we conduct is comprehensive, nuanced, and reflective of the lived experiences of the communities we aim to serve and understand. It is through this plurality of perspectives that we can challenge dominant narratives, identify gaps in knowledge, and create more effective and inclusive interventions.

**Challenges and Considerations**

We acknowledge that the work of including diverse voices is complex and ongoing. It requires us to continually reflect on our own biases, to listen deeply to the needs and suggestions of communities, and to adapt our approaches as we learn. We must be vigilant against tokenism—the superficial inclusion of diverse voices without real power or impact. Instead, we strive for meaningful engagement where all voices shape the research process and outcomes. Top of Form

Inclusive Summary:

Core Principle: We're dedicated to including a wide range of voices, especially those often overlooked, as a key ethical commitment of our research.

Valuing Diversity: Every individual's unique perspective is crucial, helping us to conduct research that truly reflects community experiences and challenges the status quo.

Active Effort: We continuously work to understand our biases, engage with community feedback, and ensure that all voices have a real influence on our research direction and findings.

Beyond Tokenism: Our goal is meaningful involvement, not just superficial diversity, to guarantee that every contribution shapes our study and its impact.

1. Bottom of Form

**Section 12: Language and Accessibility**

Language is not merely a tool for communication but also a medium through which power dynamics are expressed and perpetuated. In mutual aid work, particularly within the context of participatory research, it is imperative that we critically examine the language we use and the accessibility of our communicative practices. This awareness and deliberate action ensure that our research is inclusive and that all co-researchers can engage fully and equitably.

**Language Inclusivity**

Our choice of words, the complexity of our sentences, and the jargon we employ can either invite participation or create barriers. To foster an environment where all feel welcomed and able to contribute, we must:

- **Simplify Language**: Use plain language that can be easily understood by individuals without specialized knowledge of the research field.
- **Avoid Jargon**: Refrain from using technical terms, acronyms, or scholarly language that could alienate those not familiar with such terminology.
- **Knowledge Translate Key Documents**: Provide accessible English translations of essential documents, this could include natural readers and lay/inclusive summaries.

**Accessibility**

Accessibility in the context of participatory research extends beyond physical accommodations to encompass the full spectrum of coresearcher engagement. This includes:

- **Cultural Sensitivity and Humility**: Recognize and respect the cultural contexts and norms of all coresearchers, ensuring that our research practices do not inadvertently exclude or offend.
- **Digital Accessibility**: Ensure that all digital platforms used for the research are compatible with assistive technologies and comply with accessibility standards.
- **Flexible Engagement**: Provide various means for coresearchers to engage with the research, whether through written, verbal, visual, or other modes of communication that align with their abilities and preferences.
- **Material Accessibility**: Make sure that all materials related to the research are available in formats that are accessible to individuals with visual, auditory, or cognitive disabilities.

**Practical Application**

To operationalize these principles, we will:

- Conduct an initial assessment of coresearchers' language preferences and accessibility needs.
- Develop a glossary of terms that includes simplified explanations of technical concepts related to the research.
- Create a protocol for the adaptation of research materials, ensuring that they meet the accessibility requirements identified in the assessment.
- Establish a feedback loop where coresearchers can report issues with language or accessibility and request additional support.

**Ongoing Commitment**

Recognizing that language and accessibility needs may evolve throughout the research process, we commit to:

- Regularly revisiting and adjusting our language use and accessibility provisions to accommodate new coresearchers or changing needs.
- Actively seeking out and integrating advances in accessible communication technologies and methodologies.

Inclusive Summary

Communication Care: We're dedicated to using clear, simple language and avoiding technical jargon to ensure everyone can understand and participate fully.

Accessibility Focus: Our study is committed to accessibility in all forms, ensuring our research is available and engaging for all, regardless of physical or cognitive abilities.

Cultural Respect: We'll respect and honor the diverse cultural backgrounds of our co-researchers, adapting our methods to be culturally sensitive.

Adaptability: Recognizing diverse needs, we'll offer various ways for co-researchers to engage and provide materials in different formats.

Continuous Improvement: We pledge to continuously assess and improve our language and accessibility practices to meet evolving co-researcher needs and embrace new technologies.

**Section 13: Feedback and Evolution**

**Feedback Mechanism**

In the dynamic landscape of participatory research, the HEARTS study recognizes the importance of fostering an environment where feedback is not only welcomed but actively sought. Feedback is the cornerstone of growth and evolution within any collaborative effort. To this end, we establish a dedicated mechanism that encourages all co-researchers to share their insights, suggestions, and concerns regarding the research process and the Solidarity Statement itself.

This feedback mechanism is designed to be accessible, ensuring that every individual, regardless of their familiarity with research protocols or mutual aid concepts, can easily contribute their perspective. It will include various channels such as digital mediums, open discussion forums, and confidential one-on-one conversations, catering to the diverse preferences and needs of our co-researchers.

**Evolving the Solidarity Statement**

The Solidarity Statement is not a static document but a living agreement that reflects the collective wisdom and experiences of all co-researchers. It will be reviewed periodically, with revisions made based on the feedback received. This iterative process ensures that the Statement remains a true representation of our shared values and commitments.

The cycle of review and revision will occur every 4 months, unless urgent issues necessitate immediate attention. Each cycle will involve:

The HEARTS study is committed to embodying the principles of mutual aid not only in the work we conduct but also in the ways we interact, communicate, and grow together. The Feedback and Evolution section of our Solidarity Statement is a testament to our dedication to these principles, providing a structured yet flexible approach to ensuring our research remains a collaborative, transformative, and living endeavour.

Top of Form

Inclusive Summary:

Open Dialogue: We prioritize open communication, inviting all co-researchers to share their experiences and ideas for improvement.

Responsive Evolution: The study’s guidelines and approaches are not set in stone; they're designed to evolve based on the community's feedback.

Accessible Feedback Channels: Offering a variety of easy-to-use ways for co-researchers to voice their thoughts ensures everyone’s voice can be heard.

Regular Updates: The study’s practices and our Solidarity Statement are revisited regularly to reflect our growing understanding and the diverse perspectives of our team.

Commitment to Growth: We are dedicated to continuous learning and adaptation, ensuring our research remains relevant, inclusive, and aligned with mutual aid values.

**Section 14: Conclusion, Reflection and Commitment**

At the conclusion of our Solidarity Statement, it is crucial to reassert our collective dedication to the principles that underpin the HEARTS study. This document is not merely a set of guidelines; it represents our shared ethos, the spirit that animates our research, and the mutual commitment that binds us together as a community of co-researchers.

Mutual aid, at its core, is about reciprocal care, about showing up for each other in times of need and abundance alike. It is grounded in the understanding that our liberation is collective, that no one is free until everyone is free. The HEARTS study is a living testament to these values, and every aspect of our research is imbued with the spirit of solidarity and cooperative engagement.

In the landscape of academic research, our approach is transformative. We seek not just to observe and document, but to participate and change. We believe in the power of research to not only reflect the world, but also to shape it—to contribute to the creation of more just and equitable systems. This requires us to be more than impartial observers; it asks us to be active coresearchers in the tapestry of community life.

For those new to the concept of mutual aid, it is important to understand that this is not a new phenomenon. It is a practice as old as humanity itself, manifesting in communities coming together to support one another, without the mediation of market transactions or hierarchical structures. Mutual aid is about direct action, about meeting needs without the barriers often imposed by institutions. It is about recognizing that the well-being of the individual is inextricably linked to the well-being of the collective.

In embracing mutual aid within our research, we commit to:

- **Empathy and Compassion**: Understanding that each coresearcher brings their whole self to the study, with unique experiences and vulnerabilities that deserve to be met with empathy and compassion.
- **Interdependence**: Recognizing our interdependence and the strength that comes from relying on and supporting each other in our shared human journey.
- **Equality, Equity and Justice**: Striving for equality of all individuals being treated with dignity, worth and respect; equity in participation and justice in the outcomes of our research, ensuring that the benefits of our work extend to those who have traditionally been minoritized or silenced.
- **Active Listening and Engagement**: Fostering an environment where every voice is not only heard but actively engaged with, where we listen not to respond but to understand and learn from one another.
- **Ongoing Reflection and Adaptation**: Remaining open to change, willing to reflect on our practices, and adapt as we learn more about how to effectively embody mutual aid principles in our work.
- **Building Trust and Community**: Recognizing that trust is the foundation of any mutual aid effort and committing to the slow, deliberate work of building a community grounded in trust, respect, and shared purpose.

Our collective commitment to solidarity and mutual aid is the bedrock upon which the HEARTS study is built. It is a commitment to each other as co-researchers, to the communities we serve, and to the belief that together, we can contribute to a body of knowledge that is alive, dynamic, and transformative. Let this Solidarity Statement be a living document, one that evolves as we evolve, and stands as a testament to the power of collective action and shared dreams.

Inclusive Summary

Core Values: Our research is a collective journey guided by values of empathy, equity, and justice, deeply rooted in mutual aid principles.

Mutual Aid: This study embraces mutual aid, fostering a space where support and collaboration are not just ideas but actions we live by.

Transformative Approach: We aim to make a real impact, using research as a tool for social change and community empowerment.

Inclusive Engagement: Every voice matters in our study, and we are committed to active listening and meaningful participation.

Adaptability: We're open to growth, ready to refine our methods and practices as we learn from each other and the communities we engage with.

Trust and Community: Building a trusting community is at the heart of our work, ensuring a strong foundation for our collective research endeavours.

In solidarity and with heartfelt commitment,

[removed partners signatures for published copy]

**Key Terminology**

Respect: Acknowledgment and consideration of others' dignity and feelings, manifesting as courteous behaviour, appreciative interaction, and equitable treatment.

Inclusion: The proactive and intentional act of creating an environment in which all individuals, irrespective of their diverse attributes (race, gender, age, religion, etc.), can fully participate, contribute, and feel valued.

Equality: The state of affording all individuals equal opportunities and considerations for participation and representation, unimpeded by systemic discrimination or bias.

Acknowledgment: The act of recognizing and validating another person's experience or feelings, particularly in the context of harm or discomfort, as a precursor to restorative action.

Restorative Action: A set of steps or initiatives taken with the intent of repairing harm done and restoring trust within a community, often involving both the person who has caused the harm and the harmed party.

Trauma-Informed Principles: An approach that involves recognizing and responding to the impact of all types of trauma on individuals. It aims to create a safe and supportive environment by being aware of, and sensitive to, the emotional needs and boundaries of all coresearchers.

Trigger Warnings: A proactive statement or notation that precedes potentially distressing or traumatizing content, providing an opportunity for individuals to prepare or disengage.

Mindful Sharing: The act of consciously communicating information or experiences in a manner that minimizes emotional harm to others, taking into account both the content and context.

Emotional Labour: The process of managing one's own emotions and emotional expressions to meet the expectations of a social or professional role, often requiring significant mental effort and potential psychological cost.

Social Capital: The collective value of social networks and the inclinations arising from these networks to facilitate actions that benefit the network's members. It includes elements like trust, norms, and systems that contribute to shared values.

Intersectionality: The framework for understanding how various social identities (e.g., race, gender, class, etc.) intersect and overlap to create complex and cumulative systems of advantage or disadvantage.

Restorative Justice: A system or approach where the focus is on repairing harm through processes that involve the sufferer, the offender, and the community, seeking solutions that promote repair, reconciliation, and the rebuilding of relationships.

Transformative Justice: An approach to justice that aims not only to resolve individual conflicts or support individual sufferers but also to transform social conditions that enable the occurrence of harm and oppression.

Eudaimonia: a state of having a good indwelling spirit or being in a contented state of health, happiness, and prosperity; often used in the context of personal development and flourishing.

Flourishing: The experience of life going well, a state in which an individual experiences high levels of well-being, purpose, and effectiveness.

Thrivance: refers to the act of thriving while simultaneously making progressive advances in one's personal or community life. (This goes beyond ‘resilience’)

Glimmer: A momentary or partial view into a positive possibility or outcome, often inspiring hope or motivation; used metaphorically to describe ephemeral instances of understanding or realization.

Psychological Safety: is the belief that one can speak up, express ideas, and make mistakes without fear of punishment or humiliation in a group setting, fostering an environment of open communication and risk-taking.
