# Supplementary material for: Primus Inter PARES: First among equals—practical strategies for young adult PAtient RESearch partners (PARES) by young adult PARES
Source: Res Involv Engagem. 2024 May 8;10:45. doi: 10.1186/s40900-024-00576-0 (PMC11077772; doi:10.1186/s40900-024-00576-0)
Supplement: Supplementary file 1 — Supplementary material 1. [file 40900_2024_576_MOESM1_ESM.zip › Supplemental File - Appendix D - TransparenSCI.pdf]

October 30, 2023.

TRANSPAREN-SCI

MAPS Lab

Keeping you connected and in the loop on scientific and research activities

IN THIS ISSUE

Estimated Reading Time: 5 minutes

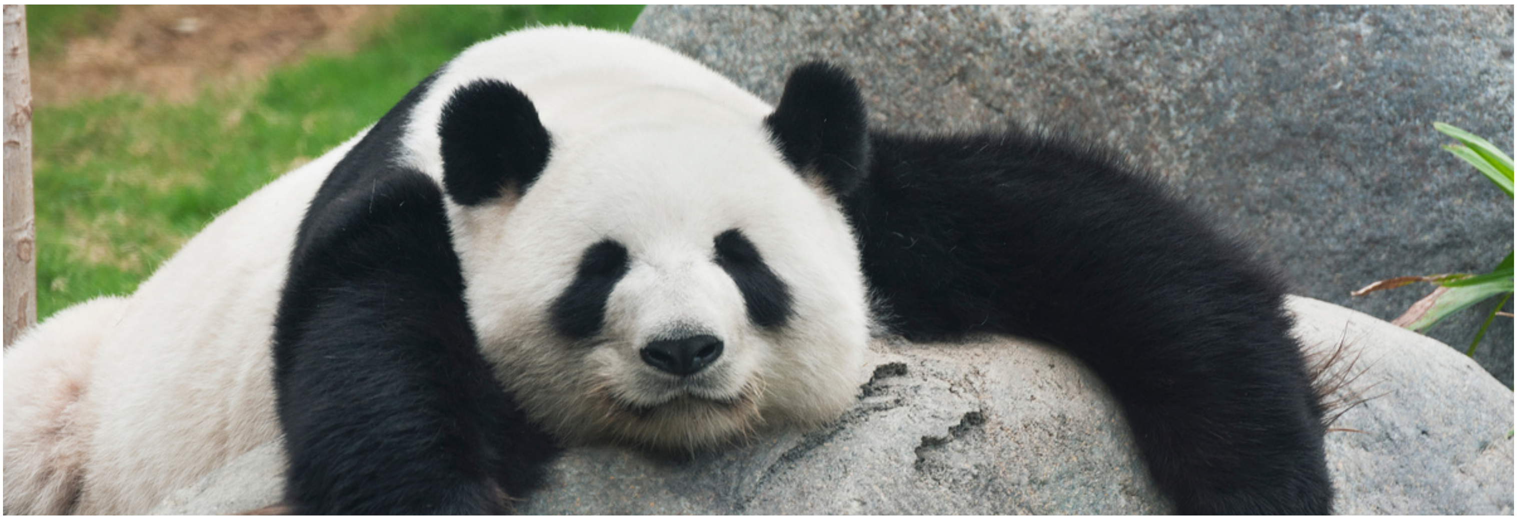

Monday's, though, am I right? For folks in the midst of mid-terms, good luck to you! The HEARTS Study team is setting time in our calendars to roll up our sleeves and get started on projects. Stay tuned for holds in your calendar.

WHAT HAPPENED LAST WEEK

We are in the process of finalizing some new members. The whole team should be onboarded within the month! Meet your new co-researcher members! Learn more about them here.

Lots of folks were interested in publishing opportunities. To learn more about academic publishing essentials, click here.

ICYMI: We are trying to apply for more funding for the patient research partners. Please join this webinar on November 14, 2023 1:00pm to 2:00pm (EST) so we can put our heads together on how to get those funds! Webex link here Password: PEG2023

This Week

WHAT'S UP FOR THIS WEEK

All Hands All Voices Gathering (aka "meeting"): Is scheduled for November 10, 2023 from 10:30am-11:30am EST (8:30am-9:30am MT) (7:30am-8:30 PT) - for folks that are not early risers, come in your PJs!

Team folders will be released by this evening. Please check your spam or junk if you do not receive an email. Action item: in your personal folder - please fill out and sign the confidentiality agreement.

Pets of the Lab: Make your pet an honourary HEARTS team member! - Send over a picture, your pet's name and what they like to do, and we'll add them to the site!

Free Workshops: Click here for some free workshops coming up.

Presentations: Do you know of any conferences that you'd like to present at? Let me know so we can keep track of the abstract submission deadlines.

Publications: Details about upcoming publication opportunities (4!) will be coming out on Wednesday; stay tuned!

Find your WAG: If you haven't done this already - fill out the WAG assessment (go to the bottom of the page to Find Your WAG) to find out where you most might like to engage in the research process. Let me know what your result was!

OPTIONAL: CLICK HERE TO PROVIDE ANONYMOUS FEEDBACK ON THE TRANSPAREN-SCI NEWSLETTER

Have a great week, everyone!
