# Supplementary material for: Primus Inter PARES: First among equals—practical strategies for young adult PAtient RESearch partners (PARES) by young adult PARES
Source: Res Involv Engagem. 2024 May 8;10:45. doi: 10.1186/s40900-024-00576-0 (PMC11077772; doi:10.1186/s40900-024-00576-0)
Supplement: Supplementary file 1 — Supplementary material 1. [file 40900_2024_576_MOESM1_ESM.zip › Supplemental Files - Appendix A - Practical Application Details.docx]

Supplemental Files – Appendix A

**Practical Application Details**

**Preparatory Work for POR**

**Accessible Blog Posts: Cultivating Participatory Research Culture**

- **Implementation**: Fifteen blog posts were initially developed to introduce PARES to participatory research and participatory research ethos from a rights and justice-oriented perspective. Blog posts are continually updated alongside any discussion item or decision point to prioritize transparency, open communication and capacity-sharing.
- **Materials and Methods**: Blogging platform, guidelines for writing in plain language.
- **Considerations**: Content relevancy and accessibility are crucial, necessitating regular updates and editorial oversight to maintain the blog's alignment with the group's culture and research progression.
- **Duration and Setup Requirements**: The initial setup of the blogging platform, learning how to use it and content creation strategy took approximately one month, with a commitment to weekly posts thereafter which take 1-2 hours per post.

**Purposive Recruitment: Targeted Engagement for Inclusivity**

- **Implementation**: Utilized a social locatedness survey based on the Canadian Community Health Survey (CCHS) to guide purposive recruitment, reflecting the population group. Initial recruitment strategies were adapted to enhance inclusivity after identifying challenges in reaching specific populations.
- **Materials and Methods**: Google Forms for survey creation, statistical software for data analysis, and outreach materials for targeted recruitment.
- **Considerations**: Proactively identifying and addressing potential recruitment biases ensures more representative participation. Creative outreach is necessary for engaging populations that are ‘hardly-reached’.
- **Duration and Setup Requirements**: Review of CCHS Data took four months, subsequent survey design and deployment took four weeks which included survey refinement. Considerable time was invested in developing outreach materials specific to the populations identified as underrepresented in the initial recruitment phase.

**Initial Meetings**

**1:1 Meetings with the IRs: Establishing Foundations of Partnership**

- **Implementation**: Initial interactions with potential PARES include comprehensive briefings on responsibilities, prioritization of health and wellness, the opportunity to design their roles, compensation, and the study’s alignment with personal values. This stage incorporates reciprocity to ensure mutual compatibility and understanding.
- **Materials and Methods**: Accessible summaries in plain language, online scheduling/booking tools for setting up conversations, mixed-media orientation packets, and video conferencing solution.
- **Considerations**: Respect for PARES' time and availability is paramount, as is providing them with thorough information to facilitate informed decision-making. IRs create availability to accommodate PARES' schedules.
- **Duration and Setup Requirements**: Approximately one month was dedicated to developing orientation materials hosted on an existing lab website, scheduling availability and setting up Zoom meetings scheduled according to PARES convenience.

**Project Responsibilities Based on Project Needs and Internal Subject Matter Expertise**

**Ethics Officer: Ensuring Ethical Vigilance in Research Practice**

- **Implementation**: A PARES ethics officer with expertise in bioethics is integrated to oversee ethical considerations, providing continuous monitoring and guidance.
- **Materials and Methods**: Frameworks and protocols for ethical decision-making in the research and with PARES and IRs.
- **Considerations**: The ethics officer must navigate a range of ethical scenarios, fostering discussions that protect IRs, PARES, and study participants and enhancing ethical awareness.
- **Duration and Setup Requirements**: The integration of the ethics officer role and the establishment of ethical guidelines took six weeks, with continuous engagement throughout the project.

**External Ombudsperson: Neutral Conflict Resolution and Support**

- **Implementation**: An impartial ombudsperson provides a confidential avenue for PARES to address concerns, ensuring fair conflict resolution and accountability.
- **Materials and Methods**: Recruiting ombudsperson with participatory experience and ability to navigate and manage conflict situations, ethics and risk, confidential reporting systems, and policy documentation for dispute resolution.
- **Considerations**: The ombudsperson's accessibility and the clarity of conflict resolution procedures are crucial to maintaining trust and fairness.
- **Duration and Setup Requirements**: The appointment of the ombudsperson and the creation of reporting mechanisms were completed within an eight-week period, with ongoing availability for PARES support.

**Research Coordinator: Administrative Leadership for Project Continuity**

- **Implementation**: Established a Research Coordinator role to oversee administrative tasks and maintain project continuity, promoting trust and accountability.
- **Materials and Methods**: Digital tools for meeting coordination, templates for authorship documentation, and organizational resources for team events.
- **Considerations**: The coordinator must demonstrate sensitivity to the diverse needs of the team while ensuring administrative tasks are completed efficiently.
- **Duration and Setup Requirements**: Recruitment, onboarding, and alignment of the Research Coordinator with project values were completed in three months, with responsibilities continually adapted to the evolving project requirements.

**Community Builder Role: Cultivating a Cohesive Research Environment**

- **Implementation**: A Community Builder was appointed to support the team in wellness, fun, and engagement activities, enhancing team culture and individual well-being.
- **Materials and Methods**: Guides for wellness activities, resources for team-building games and recreational activities, digital platforms for virtual community engagement.
- **Considerations**: Planning of activities should accommodate the varied schedules and life situations of team members without compromising research-focused meetings.
- **Duration and Setup Requirements**: The Community Builder role was established and operational within three months, with ongoing development of activities tailored to team needs.

**Rotational Publication Project Manager: Enhancing Publication Leadership**

- **Implementation**: This role is rotated among all team members per publication to manage the manuscript development and publication process, thereby diversifying leadership and enhancing the team's publication capacity.
- **Materials and Methods**: Authorship forms (e.g., Credit Taxonomy, ICJME, Conflict of Interest), publication management tools, journal selection criteria (e.g., Jane), publication templates, project management tools.
- **Considerations**: The role is structured to provide robust support and mentorship, ensuring that PARES are not left unsupported, as expressed in their prior experiences of feeling "thrown to the wolves."
- **Duration and Setup Requirements**: The rotation system for this role is implemented on a per-publication basis. The initial setup of role responsibilities and rotation processes took three months, with ongoing mentorship provided by the IRs.

**Rotating Roles and Train the Trainer: Leadership Development Dynamics** **Implementation**:

- **Implementation**: ‘Train the Trainer' model to enhance leadership skills and support a rotating roles structure within the team.
- **Materials and Methods**: Leadership development guidelines, training curricula, role rotation schedules.
- **Considerations**: Facilitate opportunities for all PARES to assume optional leadership roles, nurturing a culture of shared responsibility and collective empowerment.
- **Duration and Setup Requirements**: The development of the 'Train the Trainer' program and role rotation schedules was completed over a three month period, with continuous support and adjustment provided thereafter.

**Co-Constructing a Culture of Solidarity and Shared Values**

**Solidarity Statement: Grounding Research in Reciprocal Respect**

- **Implementation**: A dynamic solidarity statement, collaboratively drafted, articulates the research team’s shared values and commitment to mutual respect and accountability within the research process.
- **Materials and Methods**: Collaborative writing platforms, mechanisms for input and feedback, version control systems.
- **Considerations**: The statement requires periodic review and updates to remain aligned with the evolving ethos and practices of the research team.
- **Duration and Setup Requirements**: Initial discussions took three months, drafting took one month, and revisions took three weeks, with a bi-annual schedule set for revisiting and updating the document.

**Shared Values Approach: Beyond Credentials**

- **Implementation**: We prioritize shared values and passion for the research question over traditional resumes or medical diagnostic proof, fostering a values-driven research environment.
- **Materials and Methods**: Accessible materials outlining the project's values and research aims. Video conferencing for transparent and open discussions.
- **Considerations**: Articulating and communicating shared values is essential, necessitating clarity in how these values manifest within research practices.
- **Duration and Setup Requirements**: The development of value alignment research materials was completed over four weeks, with ongoing discussions to refine and communicate these values over two months.

**Consensus Building and Consent with Opt-Out Decision Making: Deliberative Inclusivity**

- **Implementation**: Employed an adapted method of consensus building, allowing PARES to opt out to mitigate cognitive load and enhance trust. This model prioritizes collective agreement over majority rule.
- **Materials and Methods**: Digital tools for consensus building and standardized opt-out processes and forms where required.
- **Considerations**: Management of the opt-out process must ensure PARES feel their autonomy is respected and their decision to opt-out is not equated with disengagement.
- **Duration and Setup Requirements**: The consensus-building tools were developed and implemented over two months with continued refinement with feedback, allowing for thorough integration into the project's decision-making processes.

**Mutual Aid Principles and Practices: Cultivating Collective Support**

- **Implementation**: Integrated mutual aid principles to foster a culture centered on collective welfare and well-being and support within the research approach.
- **Materials and Methods**: Mutual aid practice manuals, practical application guides, community-building exercise resources.
- **Considerations**: Develop mutual aid practices that are inclusive, adaptable to diverse PARES needs, and promote a supportive research environment.
- **Duration and Setup Requirements**: The process of embedding mutual aid principles into the project’s culture was ongoing, with foundational frameworks established in the initial month.

**Peer-to-Peer Buddy System: Supportive PARES Networks**

- **Implementation**: The peer-to-peer buddy system provides navigational support through the research process, fostering a network of mutual assistance among PARES.
- **Materials and Methods**: Buddy system guidelines, comprehensive contact directories, and orientation, relationship-building materials for peer-to-peer familiarization.
- **Considerations**: The system is designed to encourage supportive peer relationships while honouring individual autonomy and respecting personal boundaries.
- **Duration and Setup Requirements**: The buddy system was established at the project's outset, requiring three months for initial setup and ongoing adjustments as the team's needs evolved. The Community Builder role facilitates the development of the Peer-to-Peer Buddy System.

**Communication, Transparency and Sharing**

**Transparen-SCI Weekly Newsletter: Sustaining Connection and Transparency**

- **Implementation**: A weekly email newsletter, 'Transparen-SCI,' provides updates on the project's progress, opportunities for engagement, and pertinent developments, ensuring all PARES are kept informed.
- **Materials and Methods**: Newsletter design software, content curation material, secure distribution lists/email contact list.
- **Considerations**: The newsletter should be succinct yet comprehensive, avoiding information overload while keeping all PARES and IRs informed and connected to the research.
- **Duration and Setup Requirements**: The newsletter's framework was developed in two weeks, with content contributed by various PARES and IRs on a rolling basis.

**Capacity Sharing: A Reciprocal Learning Environment**

- **Implementation**: By emphasizing reciprocal learning, we recognize the inherent value of the knowledge each team member brings, moving away from a paternalistic 'capacity building' approach for PARES.
- **Materials and Methods**: Custom-developed training materials, interactive learning opportunities facilitated through community partners, platforms for knowledge exchange.
- **Considerations**: Learning opportunities must accommodate diverse skill levels and experiences, with a focus on accessibility and inclusivity in material design and delivery.
- **Duration and Setup Requirements**: Training material development and the setup of learning opportunities are ongoing throughout the research project to incorporate iterative feedback, testing for accessibility and progressive learning needs through the different phases of the research study.

**Growth and Continuous Improvement**

**Regular Feedback Mechanisms: Fostering Continuous Improvement**

- **Implementation**: Surveys employing quantitative and qualitative feedback mechanisms, including anonymous evaluations, are regularly distributed to gauge PARES satisfaction and research project efficacy. Additionally, PARES determine valuable metrics individually to satisfy their aspirations and goals.
- **Materials and Methods**: Easy-to-use free digital survey platforms with options for anonymity, qualitative and quantitative feedback forms.
- **Considerations**: Feedback instruments must be designed to elicit actionable insights while avoiding overburdening PARES, and maintaining anonymity and confidentiality. To maintain transparency and trust, evaluations should be shared alongside action plans for change.
- **Duration and Setup Requirements**: Survey tools were selected and customized over a three-week period, with regular feedback cycles and task-related evaluations.

**Wellness, Aspiration, and Growth (WAG) Assessment**

- **Implementation**: The WAG Assessment Tool transcends traditional skill evaluation by promoting personal development, self-acceptance, and the pursuit of individual aspirations within the research partnership.
- **Materials and Methods**: Interactive self-assessment questionnaires, engaging online platforms for tool administration, and reflective guides for personal development.
- **Considerations**: The development of the WAG Assessment Tool spanned several months, ensuring an interactive experience akin to popular online quizzes while fostering introspection and personal growth.
- **Duration and Setup Requirements**: The development, testing, and refinement of the WAG tool required a two month process to ensure its efficacy in personal development.

**Cross-Training and Reciprocal Engagement**

- **Implementation**: This approach aims to reveal hidden research skills among PARES, utilizing reflexive activities that link their life experiences with research capabilities.
- **Materials and Methods**: Guidelines for reflexive activities, workshops for cross-training, and resources connecting daily skills with research competencies.
- **Considerations**: The design of these activities was undertaken considering the PARES' diverse experiences, ensuring relevance and efficacy.
- **Duration and Setup Requirements**: The pilot phase for reflexive activities was conducted over several weeks to ensure the approach was resonant and effective.

**Resource Library: Knowledge Sharing for Empowerment**

- **Implementation**: Developed a digital resource library to provide PARES and IRs with comprehensive access to research materials, supporting their current and future growth.
- **Materials and Methods**: Content management systems for digital libraries, access protocols, and comprehensive research resources.
- **Considerations**: The library must be intuitively organized and accessible to all team members of varying research backgrounds, with materials presented in an understandable format.
- **Duration and Setup Requirements**: Compilation of the resource library spanned ten weeks, involving contributions from team members and regular updates to maintain relevance.

**Adaptive and Differential Engagement**

**Collaborative Virtual Workspaces: Sharepoint for Shared Knowledge**

- **Implementation**: Microsoft SharePoint serves as a central repository for sensitive data and resources, facilitating secure-encrypted collaboration with dedicated folders for essential documents accessible to PARES and IRs.
- **Materials and Methods**: SharePoint licenses, digital confidentiality agreements, user guides for document sharing and collaboration.
- **Considerations**: The maintenance of organized and secure virtual spaces is critical, necessitating regular updates to access controls and encryption protocols as well as backups to make certain no files are deleted.
- **Duration and Setup Requirements**: License acquisition and workspace setup were completed in three weeks, followed by ongoing management to ensure the integrity and organization of shared files, folders and resources.

**Personalized Engagement in Patient Research PARES Settings**

- **Implementation**: Our approach acknowledges the individuality of each PARES, eschewing the 'one-size-fits-all' engagement model. We tailor engagement strategies to each PARES’s unique abilities and preferences, ensuring autonomy and self-determination.
- **Materials and Methods**: Scheduling software compatible with various time zones, video conferencing tools (Zoom, Microsoft Teams, Google Meet), collaborative digital spaces (Miro, Jamboard, SharePoint), personal electronic devices (smartphones, computers), and reliable internet connections.
- **Considerations**: To accommodate diverse types of involvement, a strategy of over-recruitment was employed to ensure robust communal participation, recognizing that young adults with mental illness may require time off or time away for health and wellness needs alongside other life responsibilities. Significant time was devoted to customizing the scheduling and communication platforms to suit individual needs.
- **Duration and Setup Requirements**: The setup for personalized engagement took several weeks, with additional time allocated to train PARES on using the scheduling and communication platforms.

**Weekly Writers Circles: Fostering Academic Writing Skills**

- **Implementation**: Optional Weekly Writers Circles were instituted to support PARES in developing academic writing necessary for publication. Multiple sessions were scheduled to accommodate global time zones.
- **Materials and Methods**: Instructional materials on writing, outlines of the publication process, peer review structures, and virtual meeting environments.
- **Considerations**: Sessions were designed to cater to varying skill levels, with provisions for individualized support through structured and impromptu meetings.
- **Duration and Setup Requirements**: The implementation of Writers Circles involved organizing a schedule and creating supportive materials, adjusted continuously to meet the PARES needs.

**Public and Private PARES: Collaborative Engagement Spectrum**

- **Implementation**: Established distinct mechanisms for public and private PARES engagement, recognizing the specific needs and implications associated with various health conditions such as the stigmas associated with mental illness.
- **Materials and Methods**: Frameworks for engaging different PARES types led by PARES, agreements to ensure confidentiality, protocols for collaboration.
- **Considerations**: Transparent communication is essential to manage expectations and maintain the trust of both public and private PARES.
- **Duration and Setup Requirements**: Development of engagement frameworks took three months, including the creation of collaboration protocols.

**Diversified Communication Platforms: Streamlined Information Exchange**

- **Implementation**: Selected and optimized communication platforms tailored to different message types (e.g., data to review, informal updates) and PARES technology access levels.
- **Materials and Methods**: Selection of digital communication tools (e.g., WhatsApp, Zoom, Email, Slack), provision of necessary hardware (e.g., smartphones, computers), and solutions for reliable internet access.
- **Considerations**: Balance comprehensive communication with the potential for information saturation, while accommodating PARES' varying personal circumstances.
- **Duration and Setup Requirements**: The communication platforms were set up within the first month, with ongoing adjustments and PARES and IR training provided based on feedback.

**The Collaboratory: Co-Creative Research Space**

- **Implementation**: Developed 'The Collaboratory' as a space for collective learning and practical engagement with research data and methodologies.
- **Materials and Methods**: Collaborative virtual workspaces, data analysis and methodological tools, interactive learning environment setups.
- **Considerations**: Cultivate a comfortable and inclusive space that promotes collaborative learning and methodological innovation among all PARES.
- **Duration and Setup Requirements**: The Collaboratory was established within six weeks, with regular collective learning sessions scheduled throughout the project’s timeline.

**Sustainability and Advancing Beyond the Research Study**

**Sustainable Practices for Ongoing Community Engagement**

- **Implementation**: Fostering a supportive environment, our model focuses on maintaining engagement through community-building practices that endure beyond the project's lifespan.
- **Materials and Methods**: Tools for social networking (e.g., Whatsapp), prioritizing community building as a key goal of the participatory research process and resources for sustaining relationships.
- **Considerations**: Commitment to nurturing a supportive community required long-term planning and ongoing efforts throughout the project's duration.
- **Duration and Setup Requirements**: The establishment of a role dedicated to Community Building with a corresponding strategy took four months and is continuous throughout the research.

**Storytellers Magazine: A Platform to Reclaim Mental Health Narratives**

- **Implementation**: Established as a platform for PARES to share extensive narratives when the research environment is limited. Storytellers Magazine serves as a bidirectional channel for knowledge mobilization.
- **Materials and Methods**: Editorial standards, submission and publication infrastructure, and distribution mechanisms.
- **Considerations**: The magazine's inception took several months, focusing on creating an inclusive editorial process and supportive narrative-sharing environment.
- **Duration and Setup Requirements**: The setup involved establishing editorial guidelines and a publication pipeline to ensure a supportive platform for sharing young adult mental health experiences.

**Knowledge Mobilization: Enhancing Research Dissemination Skills**

- **Implementation**: Established a Knowledge Mobilization role to provide PARES with experience and support in public speaking and research dissemination activities.
- **Materials and Methods**: Repository of presentation resources, abstract templates, slide decks, comprehensive Q&A preparation guides.
- **Considerations**: Offer specialized support to individuals with anxiety or depression, including rehearsal opportunities and tailored mental health support mechanisms.
- **Duration and Setup Requirements**: The setup for the Knowledge Mobilization role, including the compilation of resources, was achieved within four months supported by the Canadian Strategy for Patient-Oriented Research and Research Impact Canada, with subsequent time allocated for PARES training and preparation.
